# Development of safe, effective and immunogenic vaccine candidate for diarrheagenic *Escherichia coli* main pathotypes in a mouse model

**DOI:** 10.1186/s13104-016-1891-z

**Published:** 2016-02-09

**Authors:** Asmaa Gohar, Nourtan F. Abdeltawab, Ali Fahmy, Magdy A. Amin

**Affiliations:** Viral Control Unit, National Organization of Research and Control of Biological, Cairo, Egypt; Department of Microbiology and Immunology, Faculty of Pharmacy, Cairo University, Kasr El-Aini, Cairo, 11562 Egypt; Research and Development Sector, Egyptian Company for Production of Vaccines, Sera and Drugs, The Holding Company for Biological Products and Vaccines (VACSERA), Cairo, Egypt

**Keywords:** *Escherichia coli*, Enteroaggregative, Enteropathogenic, Enteroinvasive, Enterohemorrhagic, Enterotoxigenic, Diarrhea, Immunization, Whole-cell vaccine, Formalin-killed, Interleukin 6, Interferon gamma, Adjuvant system, Alum, Cholera toxin subunit B

## Abstract

**Background:**

Enteric and diarrheal diseases are important causes of childhood death in the developing world. These diseases are responsible for more than 750 thousand deaths in children under 5 years old worldwide, ranking second cause of death, after lower respiratory diseases, in this age group. Among the major causative agents of diarrhea is *Escherichia coli*. There are several vaccine trials for diarrheagenic *E. coli*. However, diarrheagenic *E. coli* has seven pathotypes and vaccines are directed for one or two of the five main pathotypes-causing diarrhea. Currently, there are no combined vaccines available in the market for all five diarrheagenic *E. coli* pathotypes. Therefore, we aimed to develop a low-cost vaccine candidate combining the five main diarrheagenic *E. coli* to offer wide-spectrum protection. We formulated a formalin-killed whole-cell mixture of enteroaggregative, enteropathogenic, enteroinvasive, enterohemorrhagic, and enterotoxigenic *E. coli* pathotypes as a combined vaccine candidate.

**Results:**

We immunized Balb/C mice subcutaneously with 10^9^ CFU of combined vaccine candidate and found a significant increase in survival rate post challenge compared to unimmunized controls (100 % survival). Next we aimed to determine the immunological response of mice to the combined vaccine candidate compared to each pathotype immunization. To do so, we immunized mice groups with combined vaccine candidate and monitored biomarkers levels over 6 weeks as well as measured responses post challenge with relevant living *E. coli*. We found significant increase in specific systemic antibodies (IgG), interferon gamma (IFNγ) and interleukin 6 (IL-6) levels elicited by combined vaccine candidate especially in the first 2 weeks after mice immunization compared to controls (p < 0.05). We also evaluated alum and cholera toxin B subunit (CTB) as potential adjuvant systems for our candidate vaccine. We found that CTB-adjuvanted combined vaccine candidate showed significantly higher IgG and IFNγ levels than alum.

**Conclusions:**

Overall, our combined vaccine candidate offered protection against the five main diarrheagenic *E. coli* pathotypes in a single vaccine using mouse model. To the best of our knowledge, this is the first combined vaccine against the five main diarrheagenic *E. coli* pathotypes that is cost-effective with promise for further testing in humans.

**Electronic supplementary material:**

The online version of this article (doi:10.1186/s13104-016-1891-z) contains supplementary material, which is available to authorized users.

## Background

Diarrhea is a major public health problem that usually lasts a day or two and often disappears without any special treatment. However, prolonged diarrhea can cause severe dehydration and even death [1]. One of the causative agents of diarrhea is *Escherichia coli*. There are several pathotypes of *E. coli* that cause infections of the gastrointestinal system while other pathotypes cause infections outside the gastrointestinal system as bacteremia, nosocomial pneumonia and neonatal meningitis [2].

Diarrheagenic *E. coli* can be categorized into subgroups including enterotoxigenic *E. coli* (ETEC) that affects small intestine [2, 3]. ETEC is a major cause of traveler diarrhea and is responsible for 280 million diarrheal episodes and more than 400 thousand death annually [1]. Enteropathogenic *E. coli* (EPEC) affects small intestine and is responsible for infant diarrhea with fever, nausea and vomiting. Enterohaemorrhagic *E. coli* (EHEC) affects large intestine and leads to severe abdominal pain, watery diarrhea followed by bloody diarrhea leading to hemolytic uremic syndrome [2, 3]. Enteroinvasive *E. coli* (EIEC) affects large intestine and produce shigella-like diarrhea and is responsible for tissue invasion and destruction of epithelial cells [2, 3]. The fifth and final subgroup is enteroaggregative *E. coli* (EAEC), which affects small intestine and is responsible for endemic diarrhea of infants in both industrialized and developing countries [4, 5].

In diseases caused by *E. coli*, current treatments do not cure the infection, but are directed to relieve symptoms or prevent complications. Treatment includes rest and fluids administration to help prevent dehydration and fatigue. Currently researchers are investigating potential vaccines to reduce the chance of exposure to *E. coli* [6]. There are several types of vaccines including inactivated vaccines that require several additional doses or booster shots, live attenuated, subunit, toxoid, conjugate, DNA and recombinant vector vaccines [7, 8]. The development of vaccines against diarrheagenic *E. coli* pathotypes represents a major challenge because of the large number of serotypes involved and the requirement to induce immunity that is effective in the gut [9, 10].

In addition, inclusion of an immunological agent that modifies the immune response of vaccine and produce long lasting immunity is needed. These adjuvants minimize the amount of injected foreign material. Some adjuvants, such as alum are approved for human use worldwide with few exceptions. The adjuvant activity of aluminum compounds was demonstrated since 1926 with diphtheria toxoid adsorbed on alum [11]. Reports have also demonstrated that alum has limitations especially when several doses are recommended [12], so there is a need for novel model of adjuvants to be designed. Cholera toxin (CT) is a potent oral and parenteral immunogen, however, the toxicity associated with CT makes it an unlikely candidate for human use. The cholera toxin B subunit (CTB) has been used instead of cholera toxin as an adjuvant as B–subunit lacks toxicity, has potent biological properties and is a powerful mucosal and parenteral adjuvant that induces a strong immune response against co-administered or coupled antigens [13]. Another difference between CT and CTB is that CT induces the release of inflammatory cytokines such as IL-6 and IL-1*β*, while the reverse is true with CTB that inhibit IL-6 release [14].

The present work aimed to develop combined inactivated vaccine candidates of five subgroups of diarrheagenic *E.coli* to provide wide protection against different pathotypesof *E. coli*. In addition, we compared the potentials of alum and cholera toxin B subunit as adjuvants in augmenting humoral immune response to candidate *E. coli* vaccine. The results showed that candidate combined vaccine was safe and effective in protection against living *E. coli*. Thus giving promising results in stimulating humoral immune responses in mouse model. We also found that CTB elicited a significant increase in the immune response as compared with alum, suggesting better response as adjuvant.

## Results

### Mice immunized with combined candidate *E. coli* vaccine exhibited 100 % survival when challenged with living *E. coli*

**Fig. 1:**
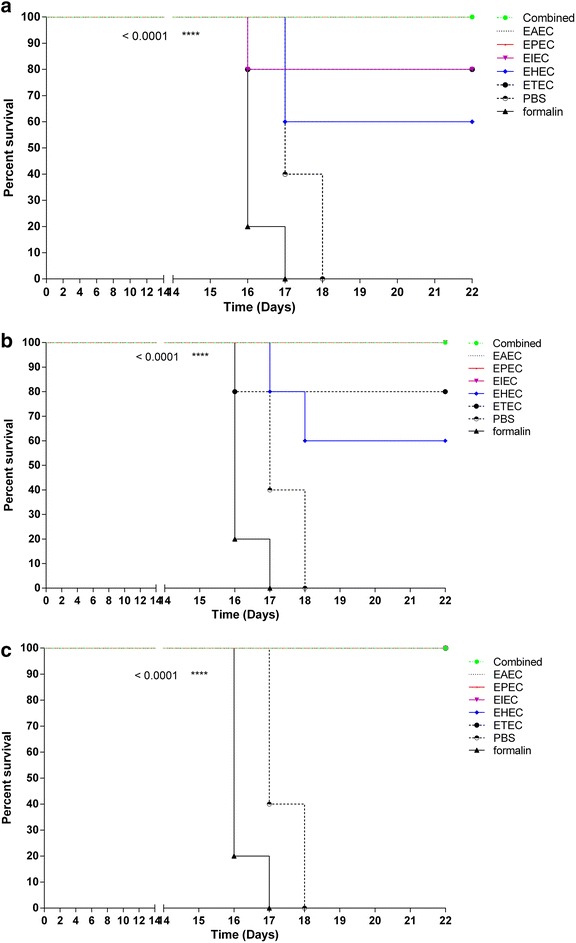
Comparative evaluation of mice survival post immunization with combined vaccine candidate versus independent individual *E. coli* antigens. Balb/C mice (n = 5 mice/group) were double immunized subcutaneously with 10^9^ CFU of formalin killed whole cell *E. coli* antigens. Antigens belonged to the main diarrheagenic *E. coli* pathotypes, Enteroaggregative *E. coli* (*EAEC*), Enteropathogenic *E. coli* (*EPEC*), Enteroinvasive *E.coli* (*EIEC*), Enterohaemorrhagic (*EHEC*) or Enterotoxigenic (*ETEC*). Combined vaccine consisted of the main five pathotypes EAEC, EPEC, EIEC, EHEC and ETEC. Two weeks later, mice were challenged intraperitoneally with 10^6^ CFU of respective living *E. coli*pathotype. Survivalcurves of mice groups post immunization with **a** unadjuvanted combined vaccine candidaterelative to unadjuvanted individual *E. coli* antigenswithvehicle (formalin) and PBS as controls. **b** Alum adjuvanted combined vaccine candidate relative to the alum adjuvanted individual antigens and controls. **c** CTB-adjuvanted combined vaccine candidate relative to the CTB-adjuvanted individual antigens using appropriate controls. *p* < 0.0001 comparing immunized groups to formalin and PBS controls

We evaluated the immune efficacy of adjuvanted and unadjuvanted formalin-killed whole-cell combined *E. coli* vaccine candidate by comparing survival of pre-immunized mice following challenge with living *E. coli*. We immunized twice Balb/C mice (n = 5 mice/group) subcutaneously with 10^9^ CFUs of unadjuvanted, alum-adjuvanted or CTB-adjuvanted formalin-killed whole-cell vaccine candidates. Combined vaccine candidate consisted of the five main pathotypes of diarrheagenic *E. coli* we formulated. We also immunized mice using the five different individual *E. coli* pathotypes, EAEC, EPEC, EIEC, EHEC, and ETEC. Formalin served as vehicle controlin addition to PBS control group, for a total of 20 groups (n = 5 mice per group) (Table 1). Two weeks after immunization, mice groups were challenged intraperitoneally with 10^6^ CFU of respective living *E. coli* pathotypesor combination of the five *E. coli*pathotypes.We found that the combined vaccine candidate whether adjuvanted or not showed 100 % survival rate post challenge compared to unimmunized controls (*p* < 0.0001) (Fig. 1). Similar responses were observed with 100 % survival ratein mice immunized with each CTB- adjuvanted *E. coli* pathotypes (Fig. 1c). However, survival rate of mice immunized with unadjuvanted or alum-adjuvanted individual *E. coli* pathotypes ranged between 50 and 100 % depending on the adjuvant (Fig. 1).

**Table 1:** *E. coli* pathotypes and mouse immunization and challenge groups used in the study

Immunization antigen (s) (whole-cell formalin-treated)	Challenge *E. coli* antigen (s) (live whole-cell)
Unadjuvanted, alum or CTB-adujvanted	
Enteroaggregative *E. coli* (EAEC) (RKI 17-2)	EAEC
Enteroinvasive *E. coli* (EIEC) ATCC 43893 (O124: NM)	EIEC
Enterohaemorrhagic *E. coli* (EHEC) ATCC 43890 (O157:H7)	EHEC
Enteropathogenic *E. coli* (EPEC) [50, 51]	EPEC
Enterotoxigenic *E. coli* (ETEC) [50, 51]	ETEC
Combined antigens cocktail: EAEC, EPEC, EIEC, EHEC, ETEC	Combined cocktail
Controls	
Formalin	Combined cocktail
PBS	Combined cocktail
	EAEC
	EPEC
	EIEC
	EHEC
	ETEC

**Fig. 2:**
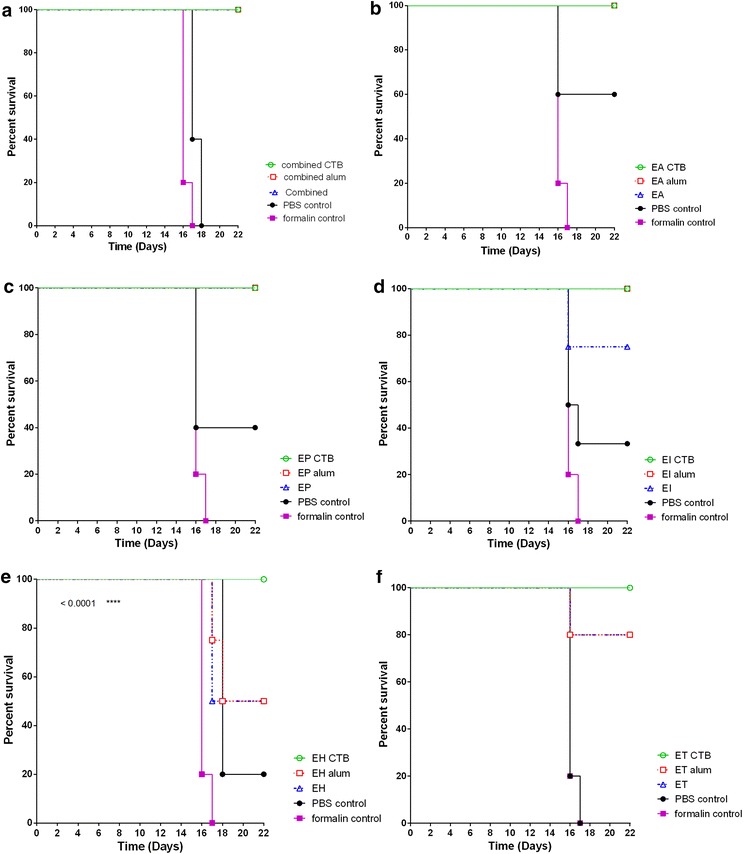
Evaluation of the effect of adjuvant system on survival rate in mice immunized with combined or individual antigens. Balb/C mice were double immunized subcutaneously with 10^9^ CFU of formalin killed whole cell antigens. Antigens belonged to the above-mentioned five-diarrheagenic *E. coli* pathotypes. Combined vaccine candidate consisted of formalin-killed whole cells of the main five pathotypes. Two weeks later, mice were challenged intraperitoneally with 10^6^ CFU of respective living *E. coli*pathotype. Evaluation of the effect of adjuvant system on the survival rate of mice groups post immunization with **a** combined vaccine candidate, **b** EAEC antigens, **c** EPEC antigens, **d** EIEC antigens, **e** EHEC antigens, and **f** EHEC antigens. In each group, appropriate controls were used. *p* < 0.05 were considered statistically significant when comparing immunized groups to formalin and PBS controls

Survival rates were different depending on adjuvant (Fig. 1) therefore we analyzed the effect of adjuvantson mice survival rate post challenge. We found that CTB showed 100 % survival compared to alum and unadjuvanted vaccine candidates (Fig. 2). For each vaccine candidate, we compared survival rates of unadjuvanted, CTB and alum-adjuvanted candidates. We found that there was no difference in survival of mice immunized with either unadjuvanted, CTB or alum adjuvanted combined vaccine candidate, with 100 % survival (Fig. 2a). Similarly, different adjuvant systems showed similar survival rates (100 %) in mice immunized with individual EAEC and EPEC pathotypes (Fig. 2b, c). However, mice immunized with CTB-adjuvanted EHEC and ETECpathotypes showed higher survival rates than alum and non-adjuvanted vaccines (Fig. 2e, f). Both adjuvant systems used offered better protection than unadjuvanted EIEC (Fig. 2d). In general, combined vaccine candidate showed 100 % survival rate irrelevant of adjuvant system used, offering protection from all five main pathotypesof diarrheagenic *E. coli* in a single dose.

We also immunized Balb/c mice using oral gavage with ~2 × 10^9^ CFU of combined vaccine. Two control groups received sodium bicarbonate vehicle and formalin orally. One week later, we challenged the mice with ~2 × 10^8^ CFU of living combined *E. coli* by oral route. We observed mice groups daily for 1 week post challenge and found that immunized mice did not develop diarrhea and survived challenge compared to controls (*p* < 0.0001) (Additional file 1: Figure S1).

### Candidate vaccine elicits specific systemic antibodies in sera of immunized mice

**Fig. 3:**
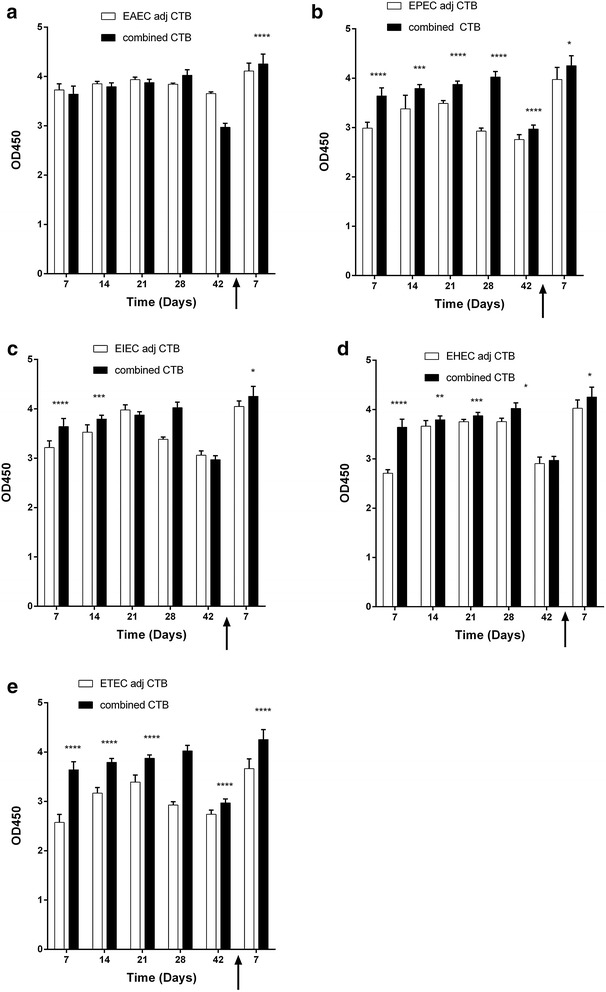
Evaluation of in vivo specific IgG antibody response measured as absorbance values elicited by CTB-adjuvanted combined vaccine candidate. Balb/C mice (n = 10 mice per group) were immunized subcutaneously with 10^9^ CFU of formalin killed whole cell antigens. Antigens belonged to the above-mentioned five-diarrheagenic *E. coli* pathotypes,. Combined vaccine candidate consisted of formalin-killed whole cell of the main five pathotypes. Post-immunization blood samples were collected from mice groups weekly for 6 weeks. At week seven, mice were challenged with 10^6^ CFU intraperitoneally and blood samples were collected 1 week after the challenge. Absorbance values of specific IgG antibody was measured for all seven intervals. Antibody absorbance values of combined vaccine candidate at selected time points compared to **a** EAEC antigens, **b** EPEC antigens, **c** EIEC antigens, **d** EHEC antigens and ETEC antigens. *p < 0.05, ** *p* < 0.001, ***p < 0.0001, and ****p < 0.00001, each *bar* represents mean ± standard deviation

With 100 % survival post challenge of immunized mice, we aimed to assess in vivo specific humoral immune responses. We used mouse model immunized subcutaneously with 10^9^ CFU of respective formalin-killed whole-cell diarrheagenic *E. coli*. Mice (n = 10 per group) were then challenged intraperitoneally with 10^6^ CFU of respective living diarrheagenic *E. coli*. Post-challenge specific IgG absorbance values against respective *E. coli* antigens were significantly higher in mice immunized with combined vaccine candidate than those immunized with individual *E. coli* pathotypes (*p* < 0.05) (Fig. 3, Additional file 2: Figure S2 and Additional file 3: Figure S3). An exception was mice immunized with EIEC alum adjuvanted and ETEC non-adjuvanted although showed higher IgG absorbance values; however did not reach significance (Additional file 2: Figure S2C and 2E).

We also monitored specific IgG antibody response over 6 weeks period post immunization with respective formalin-killed whole-cell vaccine. We selected time points 7, 14, 21, 28, 42 days to evaluate sustainability of immune response elicited by different diarrheagenic *E. coli* pathotypes in comparison to combined vaccine candidate. We found a general pattern of increase in specific IgG absorbance values compared to individual *E. coli* pathotypes at most time points (*p* < 0.05) (Fig. 3; Additional file 2: Figure S2 and Additional file 3: Figure S3). Details of exceptions to the pattern were mice immunized with CTB-adjuvanted EAEC showed comparable IgG absorbance values to combined vaccine at all time points prior to challenge (Fig. 3a). Mice immunized with alum-adjuvanted EAEC showed comparable absorbance values of IgG to combined vaccine candidate at 21, 28 and 42 days prior to challenge (Additional file2: Figure S2A) and those immunized with unadjuvanted EAEC had similar absorbance values to the combined candidate at 21 and 28 days post immunization (Additional file 3: Figure S3A). Another exception was EPEC unadjuvanted and alum adjuvanted showed comparable absorbance values of IgG to the combined vaccine at some of the time points measured (Additional file 2: Figure S2B and Additional file 3: Figure S3B). EIEC, EHEC and ETEC alum adjuvanted antigens showed comparable IgG absorbance values to combined vaccine candidate at 21, 28 and 42 h post immunization (Additional file 2: Figure S2C). In general, CTB-adjuvanted combined vaccine candidates elicited significantly higher sustained specific immunoglobulin absorbance values over 6 weeks than individual *E. coli* antigens.

### CTB-adjuvanted candidate vaccine elicits highest response of specific antibodies in sera of immunized mice

**Fig. 4:**
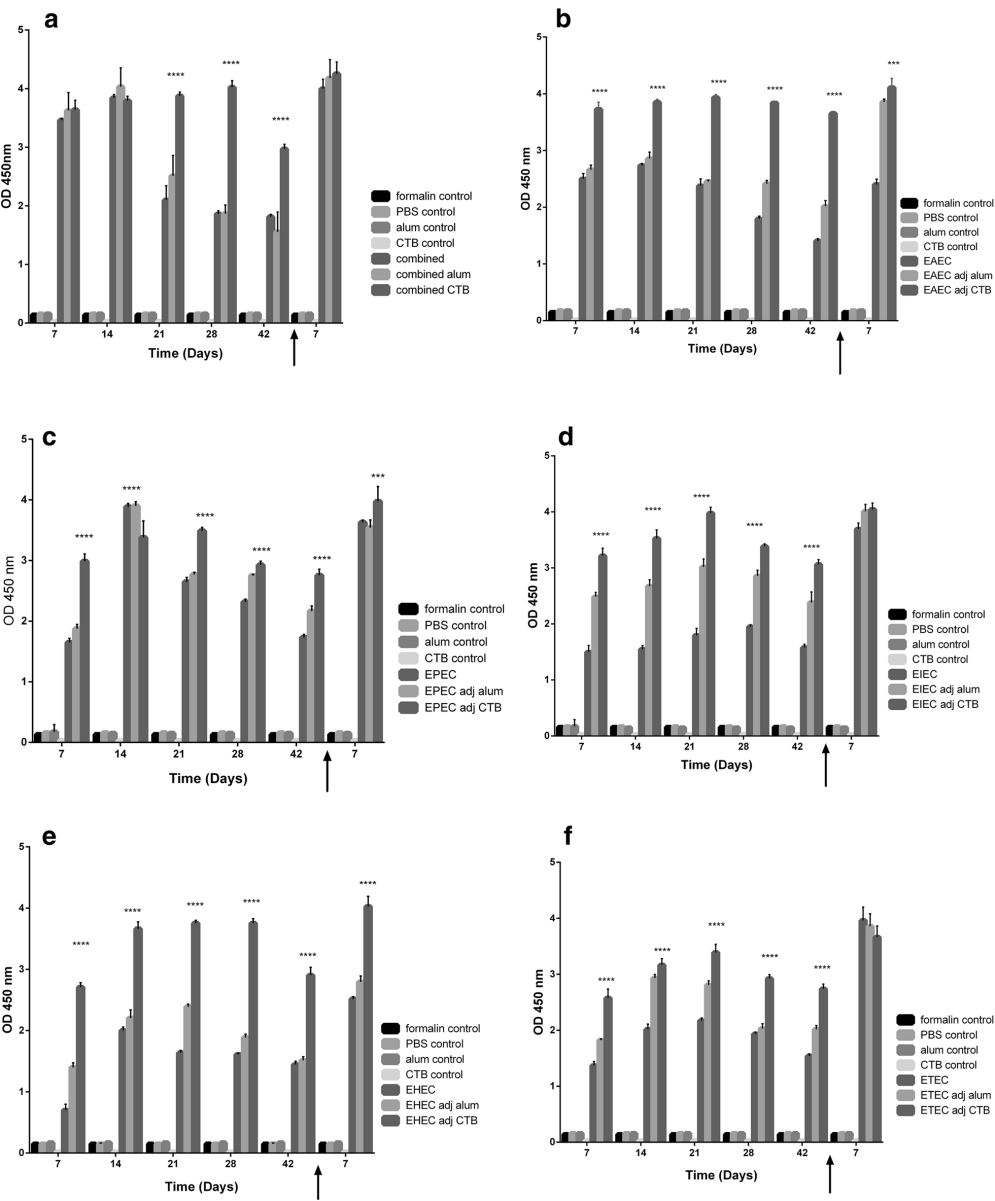
Effect of adjuvant system on antibody response measured as absorbance values of combined vaccine candidate. Balb/C mice (n = 10 mice per group) were immunized subcutaneously with 10^9^ CFU of formalin killed whole cell antigens. We used antigens of the above-mentioned five-diarrheagenic *E. coli* pathotypes. Combined vaccine candidate consisted of formalin-killed whole cell antigens of the main five pathotypes. The absorbance values of antibodies were monitored at these intervals. *Bar chart* of antibody absorbance values after mice immunization with adjuvant systems with **a** combined vaccine candidate. **b** EAEC antigens. **c** EPEC antigens. **d** EIEC antigens. **e** EHEC antigens. **f** ETEC antigens using appropriate controls. *p < 0.05, ** *p* < 0.001, ***p < 0.0001, and ****p < 0.00001, *each bar* represents mean ± standard deviation

To analyze adjuvant system immune efficacy in our combined candidate vaccine for diarrheagenic, we compared the effects of CTB and alum on absorbance values of IgG post immunization with formalin-killed whole-cell *E. coli* in Balb/C mouse model. We immunized mice with formalin-killed whole-cell *E. coli* pathotypes and combined vaccine (10^9^ CFU). We monitored serum absorbance values of IgG every week for 6 weeks post immunization. At week 7, we challenged mice with respective living *E. coli* pathotype (10^6^ CFU) and measured specific IgG absorbance values post challenge. We found in general that CTB elicited significantly higher sustained immune response over 6 weeks after immunization. When the mice were challenged with the living strains, we found increase in the immune response than before challenge with the different pathotypes. CTB achieved overall higher significance than alum and unadjuvanted vaccine candidate post challenge, but elicited comparable immune response against alum with combined candidate, EIEC and ETEC (Fig. 4a, d, f).

### Candidate vaccine stimulates pro-inflammatory cytokines production in sera of immunized mice

**Fig. 5:**
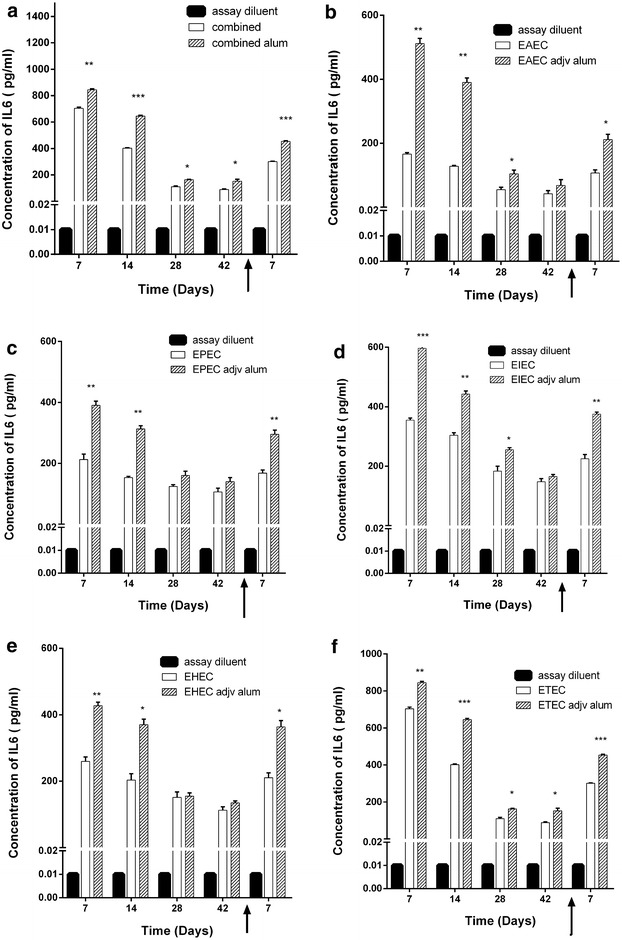
Effect of adjuvant system onIL-6 levels of combined and individual antigens. Balb/C mice (n = 10 mice per group) were immunized subcutaneously with 10^9^ CFU of formalin killed whole cell antigens. We used antigens of the above-mentioned five-diarrheagenic *E. coli* pathotypes. Combined vaccine candidate consisted of formalin-killed whole cell antigens of the main five pathotypes. The concentrations of interleukin 6 were monitored at these intervals. *Bar chart* of interleukin 6 levels after mice immunization with adjuvant systems with **a** combined vaccine candidate. **b** EAEC antigens. **c** EPEC antigens. **d** EIEC antigens. **e** EHEC antigens. **f** ETEC antigens using assay diluents as a control. *p < 0.05, ** *p* < 0.001, and ***p < 0.0001, and ****p < 0.00001 each *bar* represents mean ± standard deviation

**Fig. 6:**
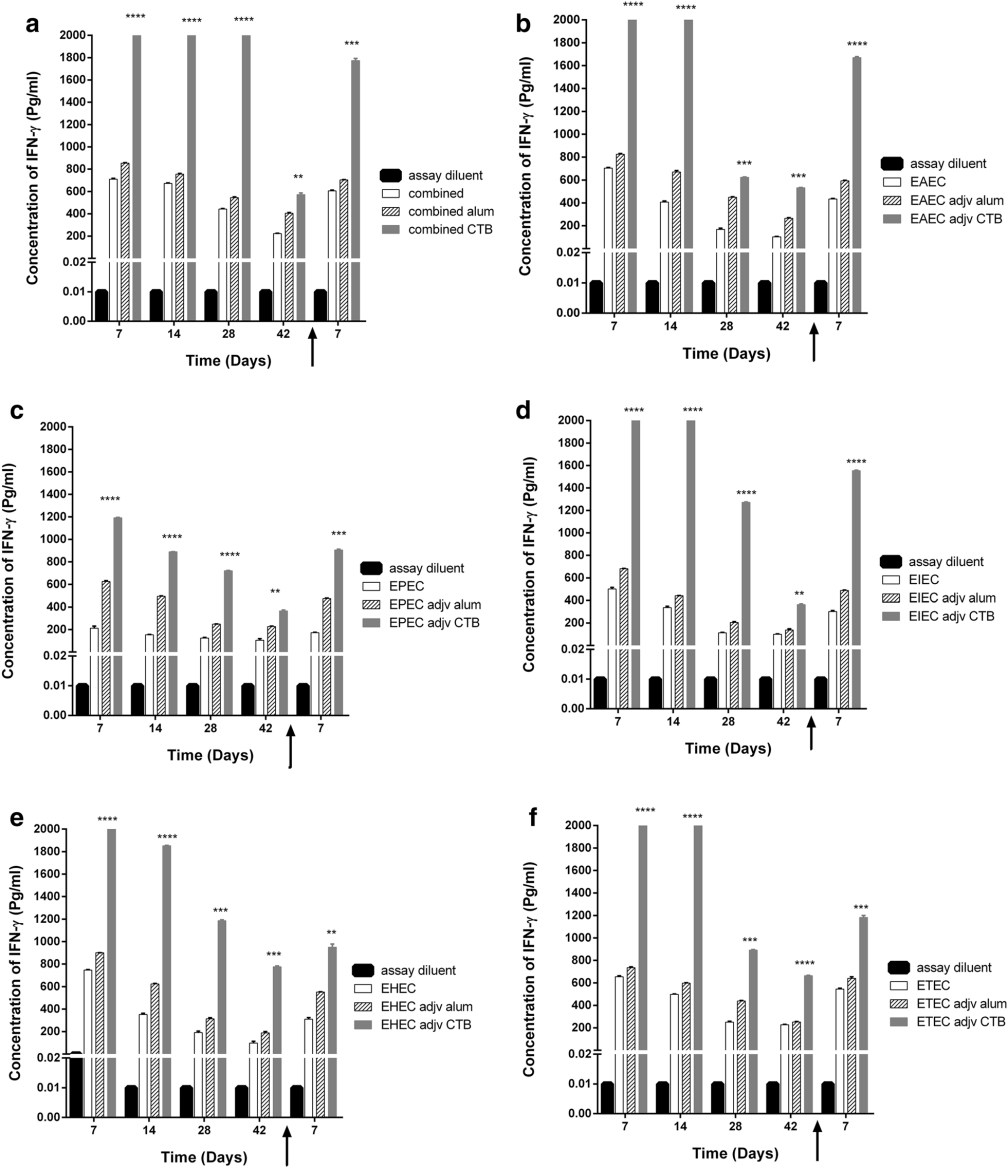
Effect of adjuvant system on IFNγ levels. Balb/C mice (n = 10 mice per group) were immunized subcutaneously with 10^9^ CFU of formalin killed whole cell antigens. We used antigens of the above-mentioned five-diarrheagenic *E. coli* pathotypes. Combined vaccine candidate consisted of formalin-killed whole cell antigens of the main five pathotypes. The concentrations of IFNγ were monitored at these intervals. *Bar chart* of IFNγ levels after mice immunization with adjuvant systems with **a** combined vaccine candidate. **b** EAEC antigens. **c** EPEC antigens. **d** EIEC antigens. **e** EHEC antigens. **f** ETEC antigens using assay diluents as a control. *p < 0.05, ** *p* < 0.001, and ***p < 0.0001, and each *bar* represents mean ± standard deviation

**Fig. 7:**
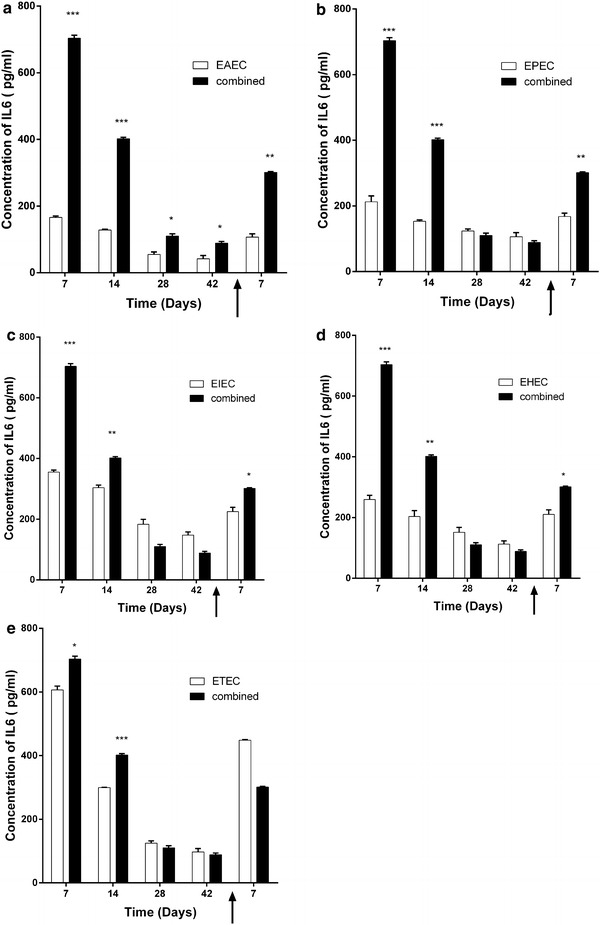
Evaluation of IL-6 levels elicited by combined vaccine candidate. Balb/C mice (n = 10 mice per group) were immunized subcutaneously with 10^9^ CFU of formalin killed whole cell antigens. Antigens belonged to the above-mentioned five-diarrheagenic *E. coli* pathotypes. Combined vaccine candidate consisted of formalin-killed whole cell of the main five pathotypes. Post-immunization blood samples were collected from mice groups weekly for 6 weeks. At week seven, mice were challenged with 10^6^ CFU intraperitoneally and blood samples were collected 1 week after the challenge. The concentration of IL-6 was measured for all seven intervals. IL-6 concentration of combined vaccine candidate at selected time points compared to **a** EAEC antigens, **b** EPEC antigens, **c** EIEC antigens, **d** EHEC antigens and **e** ETEC antigens. *p* < 0.05, ** *p* < 0.001, ***p < 0.0001, and, each *bar* represents mean ± standard deviation

**Fig. 8:**
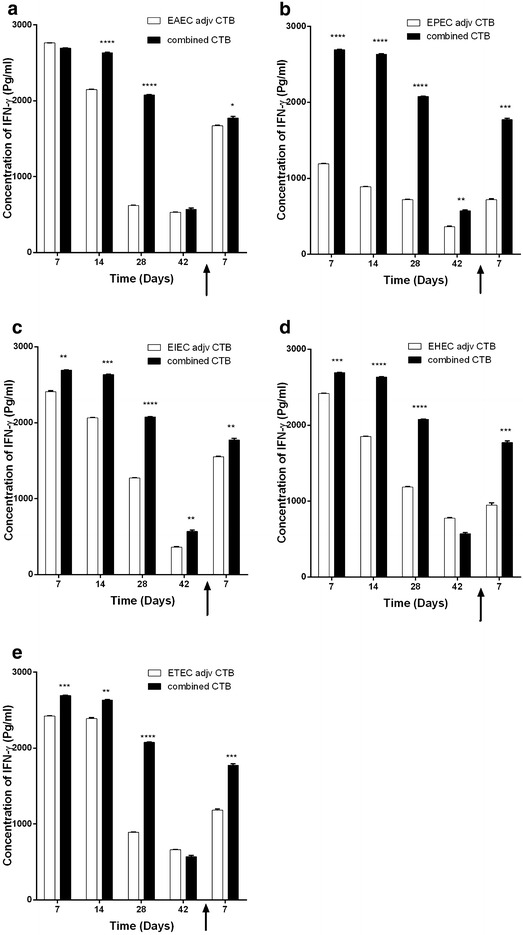
Evaluation of IFNγ levels elicited by combined vaccine candidate. Balb/C mice (n = 10 mice per group) were immunized subcutaneously with 10^9^ CFU of formalin killed whole cell antigens. Antigens belonged to the above-mentioned five-diarrheagenic *E. coli* pathotypes. Combined vaccine candidate consisted of formalin-killed whole cell of the main five pathotypes. Post-immunization blood samples were collected from mice groups weekly for 6 weeks. At week seven, mice were challenged with 10^6^ CFU intraperitoneally and blood samples were collected 1 week after the challenge. The concentration of IFNγ was measured for all seven intervals. IFNγ concentration of CTB-adjuvanted combined vaccine candidate at selected time points compared to **a** EAEC antigens, **b** EPEC antigens, **c** EIEC antigens, **d** EHEC antigens and **e** ETEC antigens. *p < 0.05, ** *p* < 0.001, and ***p < 0.0001, each *bar* represents mean ± standard deviation

We analyzed selected pro-inflammatory cytokines levels in sera of mice immunized with each pathotype and the combined five pathotypes, whether adjuvanted with CTB or alum. We found that in general, adjuvanted antigens elicit higher levels of the measured pro-inflammatory cytokines (Figs. 5 and 6). IFNγ sera levels in mice immunized with CTB-adjuvanted antigens were significantly higher than alum-adjuvanted antigens (Fig. 6). Meanwhile,IL-6 levels elicited by CTB-adjuvanted antigens were as low as diluent assay and lower than either unadjuvanted or alum-adjuvanted antigens. This is due to inhibitory effect of CTB on IL-6 production reported in several mice models [14–16]. Meanwhile, IL-6 levels elicited by candidate vaccine whether unadjuvanted or alum-adjuvanted were significantly higher than the each *E. coli* pathotype (Fig. 7 and Additional file 4: Figure S4). This is especially true during the first 2 weeks after immunization and post challenge except for ETEC (Fig. 7f). We also found that serum levels of pro-inflammatory IFNγ in mice immunized with either non-adjuvanted, CTB or alum adjuvanted were significantly higher in response to combined antigens than individual antigens (p < 0.05, 0.001 and 0.0001) (Fig. 8, Additional file5: Figure S5, and Additional file 6: Figure S6).

## Discussion

Enteric and diarrheal diseases are a major cause of childhood death in the developing world, ranking second cause of death in children under 5 years old [17]. Due to high mortality, several international organizations such as WHO and UNICEF have dedicated preventive and control programs for diarrheal diseases. Prevention methods include vaccines and improvement in water supplies, hygiene and sanitation. Among the leading causes of diarrhea is *E. coli*. Infection with various pathotypes of *E. coli* causes a high percentage of death in children under 5 years old in the developing world. ETEC diarrheal infection alone is responsible for 1.3 million-death cases annually [18].

There have been successful trials for development of vaccines to individual pathotypes of *E. coli*; these vaccine candidates are reviewed in [19–21]. Despite these successful vaccine trials, there is no single vaccine available for all the different types of *E. coli* causing diarrhea. Combination vaccines have several advantages including reducing the cost of manufacturing separate vaccines and extra health care visits. Having one combined vaccine improves timeliness of vaccination as some parents and health care providers raised objections to administering multiple injections during a single visit. This is considered a major advantage of combined vaccines [22]. Several combination vaccines have achieved great success in immunization as MMR and DTP, encouraging formulating combination vaccine for *E. coli*. Dukoral is an example of successful combined whole cell killed cholera vaccine available in market. It consists of mixture of four preparation of whole cell inactivated *Vibrio cholera* O1 (Inaba and Ogawa serotypes, classical and El Tor biotypes) with recombinant CTB [23]. We took into consideration the advantages of combined vaccines and designed our candidate-combined vaccine of different diarrheagenic *E. coli* pathotypes. Our candidate vaccine induced humoral immune response at measured time points offering protection against diarrhea in tested murine model. Mice immunized with our candidate combined diarrheagenic *E. coli* vaccine showed marked survival rate post challenge compared to control groups. In addition, high specific antibodies responses to each antigen were elicited post immunization of mice and at selected time points and post challenge.

There is a possible relationship between the maintenance of specific antibody response post immunization with inactivated whole-cell *E. coli* mixture and B cell mediated memory response to challenge with living bacteria [21, 24]. This is evident in our study where, stimulation of the immune response via immunization with combined vaccine containing inactivated whole-cells of the five main *E. coli* pathotypes led to production of specific IgG in immunized mice. This specific IgG response was sustained over the period of 6 weeks post immunization (Fig. 4). Due to specific B-cell memory immune responses, this specific IgG was significantly high post challenge. Specific IgG response in combination with production of IFNγ conferred protection from diarrhea (Figs. 4, 6, 8). Thus resulting in significantly higher survival (100 %) of the immunized mice compared to controls (P < 0.005) (Fig. 2). Adjuvant systems used in this study, alum and CTB, have potentiated action of the antigen. Due to variations in adjuvant nature of alum and CTB, where CTB in itself is immunogenic, thus induced higher immune response than alum post immunization. In addition, CTB maintained a good level of specific antibodies than alum (Fig. 4). Mean while, post challenge, there was no significant difference between CTB and alum as adjuvant systems in the specific IgG response except in EHEC, EAEC and EPEC where CTB induced the highest response (Fig. 4). This might be due to the excellent adjuvant effect of both CTB and alum, however the exact reason might need further investigation.

We also demonstrated that there was a significant increase in relevant cytokines levels post immunization. These are important features to any effective vaccine candidate where specific response was attained. Developments of vaccines against various pathotypes of *E. coli* using animal models have usually used specific subunits, toxins, secreted, or recombinant proteins offering significant protection [25–30]. The use of specific subunits, toxins or recombinant proteins offers significant protection and has been critical in recent advances in vaccines development. However, we chose to use whole–cell vaccine due to several reasons. One of these reasons is that preparation of whole-cell vaccines is easy and of low-cost, making such a vaccine cost-effective and very useful for poor countries where diarrhea is a critical health problem among infants and children [31, 32]. Additionally, oral ETEC whole-cell vaccines have been proven safe and effective in immunizing infants and children in clinical trials. In addition, oral whole-cell typhoid vaccine, Ty2a, has proved to be effective, safe and immunogenic offering protection from typhoid. Moreover, we aimed to offer wide-protection by combining major pathotypes of diarrheagenic *E. coli* and having all antigens of a whole-cell vaccine. Thus, our candidate combined vaccine is simple to prepare in addition it offers both efficacy and cost-effectiveness.

Dosing is another important consideration when formulating *E. coli* vaccines. Usually several doses are needed to maintain an efficient immunity. This would be an economical burden for developing countries where diarrhea caused by *E. coli* is prevalent. To overcome this limitation, vaccines are formulated with adjuvants that stimulate the immune response at either the cellular or humoral level and generate a depot effect, by stimulating a granuloma around the antigen. However, the choice of an appropriate adjuvant requires careful considerations [33]. One of the commonly used adjuvants in human vaccination is alum. Alum is found in numerous vaccines, including diphtheria-tetanus-pertussis, human papillomavirus and hepatitis vaccines [34]. Alum provokes a strong Th2 response, but it is rather ineffective against pathogens that require Th1–cell-mediated immunity [35]. However, alum has several side effects when high doses of vaccines are required. High levels of alum in the body affect multiple organs causing brain and bone tissue damage leading to fatal neurological syndrome [35]. Another commonly used adjuvant is cholera toxin (CT). CT has immune modulatory effects on different cell types; the interaction of CT with dendritic cells may be critical for its adjuvant activity [36, 37]. In addition, CT augments cellular immune responses of co-administrated antigens eliciting the production of IL-4, IL-5 and IL-10 [38]. B-subunits of CT are the commonly used form of CT in enhancing the immune response to conjugated antigens [38–44]. All these immunogenic effects of adjuvants make their choice an important consideration in vaccine development. Therefore, another aim of our work was to study the effect of adjuvants on the immune response of whole cell formalin killed diarrheagenic.

Our results showed a significant increase in the immune responses associated with the whole cell formalin inactivated *E. coli* combined with cholera toxin B subunit than that achieved with alum. IFNγ levels increased after immunization whether using CTB or alum in agreement with previous studies [45, 46]. However, we found that CTB-adjuvanted vaccine candidates elicited a significant increase in IFNγ level than that detected with alum. The reverse was observed withIL-6, where alum increased the concentration of IL-6, but CTB did not affect its secretion in agreement with [14–16]. This is due to possible inhibitory effects of CTB on both B cell dependent and independent pathways for IL-6 production [15, 16].

Survival rate after mice challenge is used to evaluate the efficacy and safety of the formulated vaccine candidates. Our results showed that the survival rate after immunization with vaccine candidate whether non-adjuvanted or adjuvanted was significantly higher than the controls. However, on comparing selected adjuvants, we found that survival rate post immunization with CTB-adjuvanted vaccine was higher than alum. Collectively, our results suggested that CTB-adjuvanted combined vaccine had more advantages than alum adjuvanted as far as protection, eliciting immune responses and survival rates.

Finally, we aimed to assess involvement of immune cell populations in protection, we analyzed cell populations post immunization and challenge in mice groups. Different *E. coli* pathotypes have been reported to induce mucosal inflammatory responses with infiltration of neutrophils to site of infection [47–49]. However, in our model of combined vaccine candidate, we found that NLR (neutrophil lymphocyte ratio) did not significantly differ from the controls and this is in agreement with [50] where killed bacterial vaccines usually do not elicit migration of neutrophils.

## Conclusions

We developed and tested a new combined vaccine in mice, alone and with an adjuvant system. The vaccine contained *E. coli* main five diarrheagenic pathotypes ETEC, EHEC, EIEC, EAEC and EPEC. Immunization of mice induced serum antibody responses to all antigens in the combined vaccine. In addition, immunized mice had protective pro-inflammatory responses and higher survival rates post challenge. These responses were further enhanced by adjuvant systems whether alum or CTB. We found that CTB was a better adjuvant to our vaccine candidate than alum. The vaccine, both with and without adjuvants, was stable and well tolerated. Our ongoing studies focuses on formulating our candidate vaccine as oral instead of subcutaneous vaccine. A change in route of vaccine administration is anticipated to be successful as there have been several oral vaccine trials that proved safe and immunogenic using various *E. coli* diarrheagenic pathotypes, for example ETEC/rCTB [31, 32]. Our candidate vaccine offers protection against the five main diarrheagenic *E. coli* in a single dose. However, vaccination might affect gut flora in human beings and it would be interesting to see if our candidate combined vaccine might affect gut microbiota prior to clinical trials. In addition, further studies on extended immunity and clinical studies in humans would be valuable.

## Methods

### Bacterial strains and culturing conditions

We used standard reference strains of Enteroaggregative *E.coli* (EAEC) (RKI 17-2), Enteroinvasive *E. coli* (EIEC) ATCC 43893 (O124:NM, USA), and Enterohaemorrhagic *E. coli* (EHEC) ATCC 43890 (O157:H7). In addition, reference strains of Enterotoxigenic *E. coli* (ETEC) and Enteropathogenic *E. coli* (EPEC) were kindly provided by Dr. Marwa E.A. Aly [51, 52].

*Escherichia coli* were cultured in Luria Broth (LB) (Difco, Sparks, MD, USA) at 37°c for 14 h and harvested with phosphate buffer saline (PBS) (Bio Basic Inc., Ontario, Canada). Cells were washed in PBS by centrifugation at 500×g for 10 min at 4°c [53]. The concentration of *E. coli* was adjusted at 10^9^ CFU/ml. Formalin (Fischer-scientific, Leicestershire, UK) was then added (0.4 %) to kill *E. coli.* The formalin treated bacteria was kept for 24 h to ensure killing action of the formalin. Formalin-treated bacterial suspensions were confirmed dead as there was no living microorganisms found on inoculating the suspensions in LB. The killed organisms were harvested by centrifugation at 6000×g for 1 h at 4°c then washed twice with sterile PBS (pH 7.2). The pellets obtained were re-suspended in PBS. We performed sterility testing of prepared formalin inactivated vaccine to check if there was contamination by other bacteria. Sterility test was done by taking a loop full of the prepared formalin killed antigen onto blood agar, McConkey agar plates and thioglycollate broth (Sigma Aldrich, Saint Louis, MO, USA) and incubating for 24–48 h at 37° [54].

### Adjuvants preparation

We prepared Aluminum phosphate (alum) (Sigma Aldrich, Saint Louis, MO, USA) by preparing 0.63 M AlCl_3_.6H_2_O and sodium phosphate solution(0.3 M Na_3_PO_4_.12 H_2_O)each in 40 ml saline and sterilized by filtration. Final alum adjuvant was prepared according to the method detailed in [12, 55, 56]. Recombinant CTB (rCTB) was kindly provided from the Holding Company for Biological Products and Vaccines (VACSERA, Giza, Egypt).

### Animals

Balb/C male and female mice, 6–8 weeks old were used for all experiments. The mice were purchased from VACSERA to Helwan (VACSERA vivarium, Helwan, Egypt). Animals were housed in accordance with standard laboratory conditions with access to food and water ad libitum, in an environmentally controlled room with 12 h light and dark cycles. A total of 325 mice were used in this study to determine effect of combined candidate vaccine on survival post-challenge and immunological response of mice to the candidate vaccine.

All animal experiments were conducted in accordance with the institutional regulations. The Institutional Ethical Committee of the Faculty of Pharmacy, Cairo University, Egypt, approved animal studies (approved protocol number MI (737)).

### Mice immunizations

Balb/C mice (n = 5 mice/group, for a total of 125 mice) were double immunized one-week apart subcutaneously with 10^9^ CFU of respective unadjuvanted, alum–adjuvanted or CTB-adjuvanted formalin–killed whole–cell combined vaccine candidate (Table 1). We also immunized the mice with each of the five main diarrheagenic *E. coli* pathotypes, EAEC, EPEC, EIEC, EHEC, and ETEC. Formalin served as vehicle control in addition to PBS control group, for a total of 25 groups (Table 1). After 2 weeks the mice were injected the challenge dose 10^6^ CFU (0.5 ml) of respective *E. coli* pathotype intraperitoneally and the mice were observed for 7 days and the rate of survival was determined [57].We tested our candidate vaccine using oral route by immunizing Balb/c mice by oral gavage with ~2 × 10^9^ CFU of combined vaccine in 300 μl of sodium bicarbonate twice 1 week apart. Two control groups received orally 300 μl of sodium bicarbonate vehicle and formalin. We observed mice daily for 1 week then the mice were challenged with ~2 × 10^8^ CFU of living combined *E. coli* pathotypes by oral route. We observed mice groups daily for 1-week post challenge.

### Assessment of immunological response patterns of the candidate vaccine

We immunized Balb/C mice (n = 10 per group) subcutaneously with 10^9^ CFU of respective formalin-killed whole-cell diarrheagenic *E. coli* pathotypes (Table 2). We collected blood from mice to monitor the immune responses weekly for 6 weeks. At week 7, mice were then challenged intraperitoneally with 10^6^ CFU of respective living diarrheagenic *E. coli* pathotype and monitored for one-week post challenge. Control groups included CTB, alum, formalin and PBS (n = 5 per control group), for a total of 200 mice used in immunological response assessment experiments. All control groups were challenged with combined candidate vaccine (Table 2).

**Table 2:** Groups of mice used in assessment of immunological response to candidate vaccine

Immunization antigen	Challenge *E. coli* pathotype
Unadjuvanted, alum or CTB-adjuvanted	
Combined	Combined
EAEC	EAEC
EPEC	EPEC
EIEC	EIEC
EHEC	EHEC
ETEC	ETEC
Controls	
Formalin	Combined
PBS	Combined
Alum	Combined
CTB	Combined

### Detection of antibodies against *E. coli* antigen

We collected blood samples weekly throughout the experiment via retro-orbital plexus of immunized mice groups (Table 2). Sera were collected by cold centrifugation for 15 min at 5000 rpm. Enzyme Linked Immunosorbent assay (ELISA) plates (Nunc-Denmark) were coated with whole-cell inactivated *E. coli* antigen in carbonate-bicarbonate buffer (Sigma Aldrich, Saint Louis, MO, USA), pH 9.6 as 100 µl/well(6 µg/ml) and the experiment was processed according to established previous studies [58–60]. Sera from two mice per group were pooled and assessed for a total of five mice pools per group.

### Cytokines assays

We measured levels of pro-inflammatory cytokines in sera of mice groups (Table 2).We pooled sera of five mice per group prior to assay and assessed levels of IL-6 and IFNγ using ELISA method according to the manufacturer’s protocol (BiooScientific Co., Austin, TX, USA). Concentrations were calculated based on standard curve analysis (Additional file 7: Figure S7).

### Complete blood count analysis

We collected blood from mice in EDTA-coated tubes and analyzed percentages of neutrophils and lymphocyte using slide method. We calculated neutrophils lymphocyte ratio. All samples were compared to the control groups.

### Statistical analysis

Data were analyzed using GraphPad Prism 6.01 (GraphPad Software Inc., California, USA). We used analysis of variance (one way analysis of variance (ANOVA)) for comparison of cytokine and antibody levels means. Statistical analyses for comparison of antibody absorbance values or cytokine levels elicited by combined candidate vaccine and each of *E. coli* antigens was calculated using unpaired Student’s t-test. Significance testing for comparison of survival curves was done using Log-rank (Mantel- Cox)test. *p* less than 0.05 were considered significant.

## Additional files

10.1186/s13104-016-1891-z Comparative evaluation of mice survival post immunization with combined vaccine candidate versus controls. Balb/C mice (n = 5 mice/group) were immunized twice (a week apart) by oral route with 10^9^ CFU of formalin killed whole cell combined candidate of diarrheagenic *E. coli*. Combined vaccine consisted of the above-mentioned five-diarrheagenic *E. coli* pathotypes. Two weeks later, mice were challenged orally with 10^8^ CFU of live combination of diarrheagenic *E. coli* pathotypes. Survival curves of mice groups post immunization with unadjuvanted combined vaccine candidate relative to formalin and sodium bicarbonate controls. *p* < 0.05 was considered significant, **** *p* < 0.0001 comparing immunized groups to formalin and sodium carbonate controls.
10.1186/s13104-016-1891-z Evaluation of in vivo specific IgG antibody response measured as absorbance values elicited by alum adjuvanted combined vaccine candidate. Balb/C mice (n = 10 mice per group) were immunized subcutaneously with 10^9^ CFU of formalin killed whole cell antigens. Antigens belonged to the above-mentioned five-diarrheagenic *E. coli* pathotypes. Combined vaccine candidate consisted of formalin-killed whole cell of the main five pathotypes. Post-immunization blood samples were collected from mice groups weekly for six weeks. At week seven, mice were challenged with 10^6^ CFU intraperitoneally and blood samples were collected one week after the challenge. Absorbance value of specific IgG antibody was measured for all seven intervals. Antibody absorbance values of combined vaccine candidate at selected time points compared to **A)** EAEC antigens, **B)** EPEC antigens, **C)** EIEC antigens, **D)** EHEC antigens and **E)** ETEC antigens. *p* < 0.05, ****p* < 0.0001, and *****p* < 0.00001, each bar represents mean ± standard deviation.
10.1186/s13104-016-1891-z Evaluation of in vivo specific IgG antibody response measured as absorbance values elicited by unadjuvanted combined vaccine candidate. Balb/C mice (n = 10 mice per group) were immunized subcutaneously with 10^9^ CFU of formalin killed whole cell antigens. Antigens belonged to the above-mentioned five-diarrheagenic *E. coli* pathotypes. Combined vaccine candidate consisted of formalin-killed whole cell of the main five pathotypes. Post-immunization blood samples were collected from mice groups weekly for six weeks. At week seven, mice were challenged with 10^6^ CFU intraperitoneally and blood samples were collected one week after the challenge. Absorbance values of specific IgG antibody were measured for all seven intervals. Antibody absorbance values of combined vaccine candidate at selected time points compared to **A)** EAEC antigens, **B)** EPEC antigens, **C)** EIEC antigens, **D)** EHEC antigens and **E)** ETEC antigens. (**p* < 0.05, ** *p* < 0.001, ****p* < 0.0001, and *****p* < 0.00001), each bar represents mean ± standard deviation.
10.1186/s13104-016-1891-z Evaluation of IL-6 levels elicited by alum adjuvanted combined vaccine candidate. Balb/C mice (n = 10 mice per group) were immunized subcutaneously with 10^9^ CFU of formalin killed whole cell antigens. Antigens belonged to the above-mentioned five-diarrheagenic *E. coli* pathotypes. Combined vaccine candidate consisted of formalin-killed whole cell of the main five pathotypes. Post-immunization blood samples were collected from mice groups weekly for six weeks. At week seven, mice were challenged with 10^6^ CFU intraperitoneally and blood samples were collected one week after the challenge. The concentration of IL-6 was measured for all seven intervals. IL-6 concentration of combined vaccine candidate at selected time points compared to **A)** EAEC antigens, **B)** EPEC antigens, **C)** EIEC antigens, **D)** EHEC antigens and **E)** ETEC antigens. **p* < 0.05, ** *p* < 0.001, and ****p* < 0.0001 each bar represents mean ± standard deviation.
10.1186/s13104-016-1891-z Evaluation of IFNγ levels elicited by alum adjuvanted combined vaccine candidate. Balb/C mice (n = 10 mice per group) were immunized subcutaneously with 10^9^ CFU of formalin killed whole cell antigens. Antigens belonged to the above-mentioned five-diarrheagenic *E. coli* pathotypes. Combined vaccine candidate consisted of formalin-killed whole cell of the main five pathotypes. Post-immunization blood samples were collected from mice groups weekly for six weeks. At week seven, mice were challenged with 10^6^ CFU intraperitoneally and blood samples were collected one week after the challenge. The concentration of IFNγ was measured for all seven intervals. IFNγ concentration of combined vaccine candidate at selected time points compared to **A)** EAEC antigens, **B)** EPEC antigens, **C)** EIEC antigens, **D)** EHEC antigens and **E)** ETEC antigens.**p* < 0.05, ** *p* < 0.001, and ****p* < 0.0001, each bar represents mean ± standard deviation.
10.1186/s13104-016-1891-z Evaluation of IFNγ levels elicited by unadjuvanted combined vaccine candidate. Balb/C mice (n = 10 mice per group) were immunized subcutaneously with 10^9^ CFU of formalin killed whole cell antigens. Antigens belonged to the above-mentioned five-diarrheagenic *E. coli* pathotypes. Combined vaccine candidate consisted of formalin-killed whole cell of the main five pathotypes. Post-immunization blood samples were collected from mice groups weekly for six weeks. At week seven, mice were challenged with 10^6^ CFU intraperitoneally and blood samples were collected one week after the challenge. The concentration of IFNγ was measured for all seven intervals. IFNγ concentration of combined vaccine candidate at selected time points compared to **A)** EAEC antigens, **B)** EPEC antigens, **C)** EIEC antigens, **D)** EHEC antigens and **E)** ETEC antigens. **p* < 0.05, ** *p* < 0.001, and ****p* < 0.0001, each bar represents mean ± standard deviation.
10.1186/s13104-016-1891-z Standard curve of IL-6 and IFNγ in pg/ml.

## Abbreviations

ANOVA: analysis of variance
CFU: colony forming unit
CT: cholera toxin
CTB: cholera toxin subunit B
DNA: deoxyribonucleic acid
EAEC: enteroaggregative *Escherichia coli*
EDTA: ethylenediaminetetraacetic acid
EHEC: enterohemorrhagic *Escherichia coli*
EIEC: enteroinvasive *Escherichia coli*
ELISA: enzyme Linked Immunosorbent assay
EPEC: enteropathogenic *Escherichia coli*
ETEC: enterotoxigenic *Escherichia coli*
IFNγ: interferon gamma
IgG: immunoglobulin G
IL: interleukin
PBS: phosphate buffer saline
rCTB: recombinant cholera toxin subunit B
