# Supplementary figures and images for: Development of safe, effective and immunogenic vaccine candidate for diarrheagenic Escherichia coli main pathotypes in a mouse model
Source: BMC Res Notes. 2016 Feb 9;9:80. doi: 10.1186/s13104-016-1891-z (PMC4748553; doi:10.1186/s13104-016-1891-z)

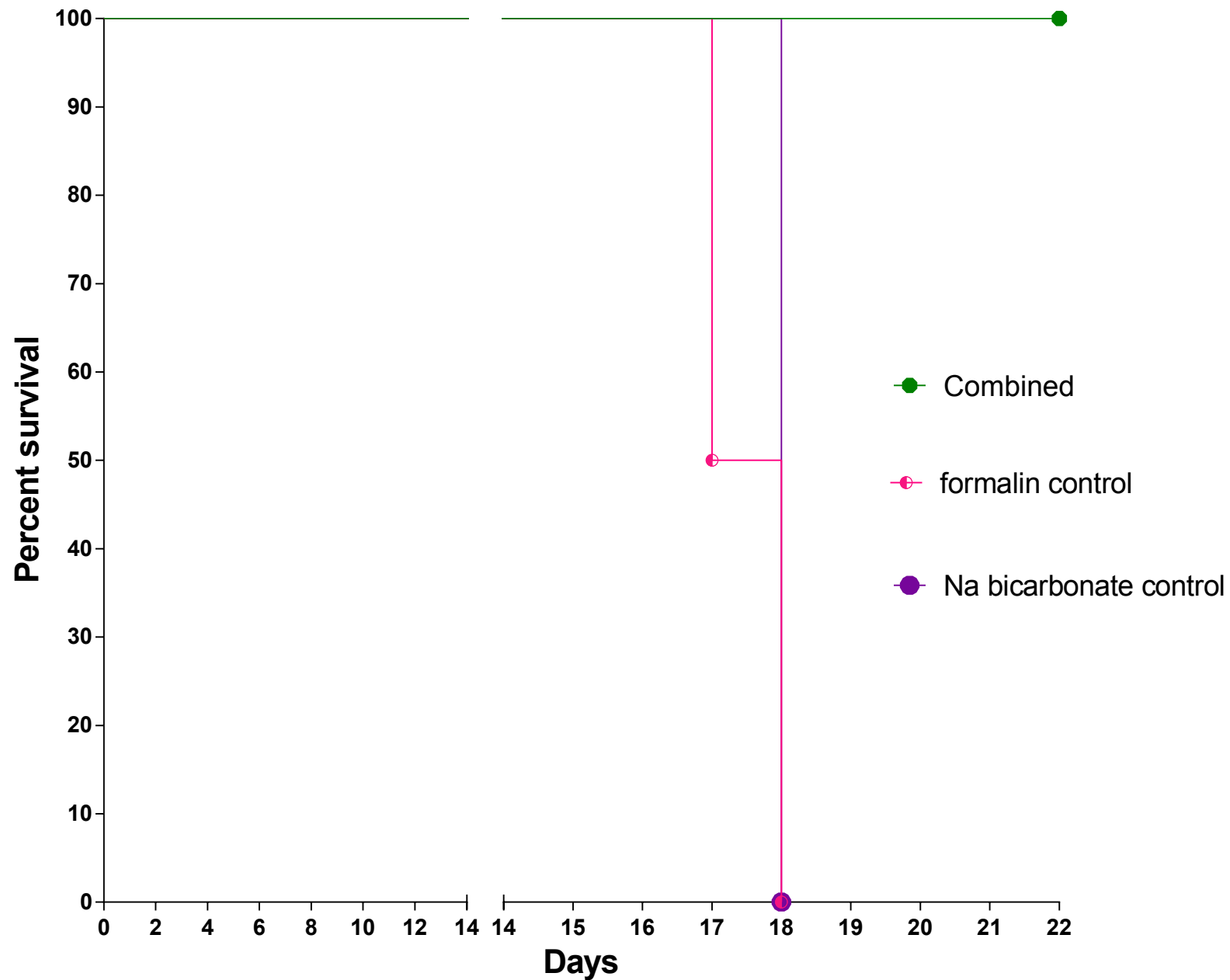

Supplement: Supplementary file 1 — 10.1186/s13104-016-1891-z Comparative evaluation of mice survival post immunization with combined vaccine candidate versus controls. Balb/C mice (n = 5 mice/group) were immunized twice (a week apart) by oral route with 109 CFU of formalin killed whole cell combined candidate of diarrheagenic E. coli. Combined vaccine consisted of the above-mentioned five-diarrheagenic E. coli pathotypes. Two weeks later, mice were challenged orally with 108 CFU of live combination of diarrheagenic E. coli pathotypes. Survival curves of mice groups post immunization with unadjuvanted combined vaccine candidate relative to formalin and sodium bicarbonate controls. p < 0.05 was considered significant, **** p < 0.0001 comparing immunized groups to formalin and sodium carbonate controls. [file 13104_2016_1891_MOESM1_ESM.pdf]

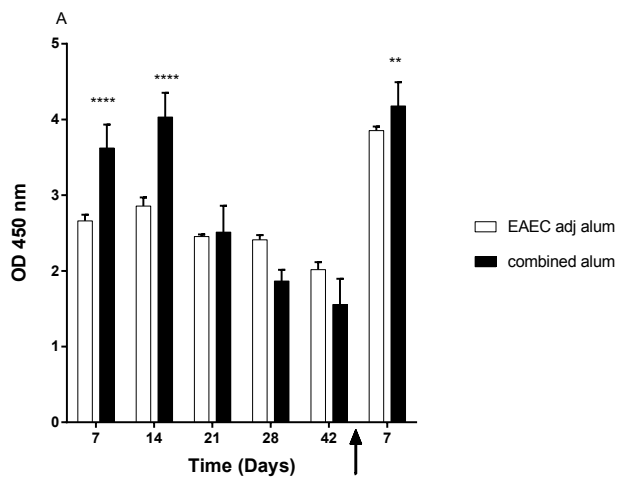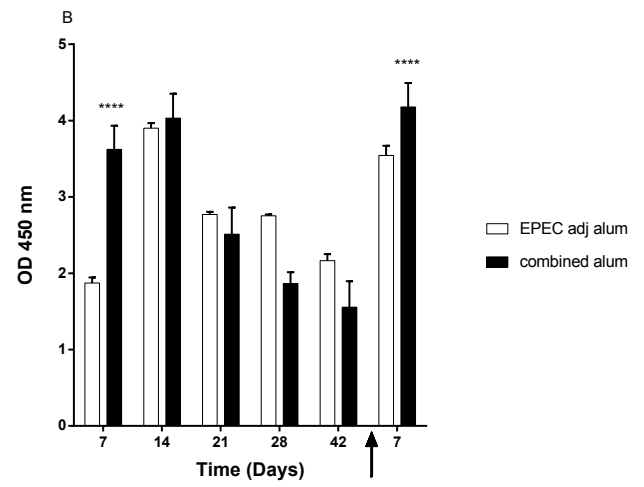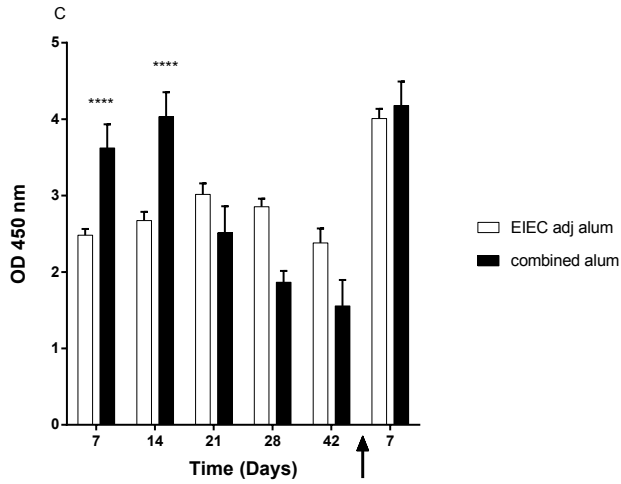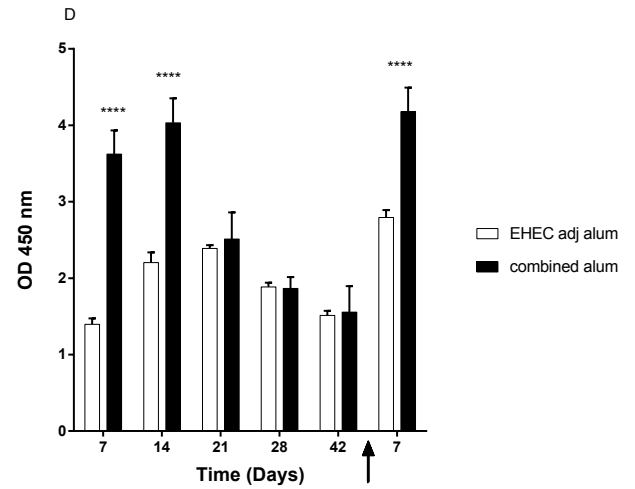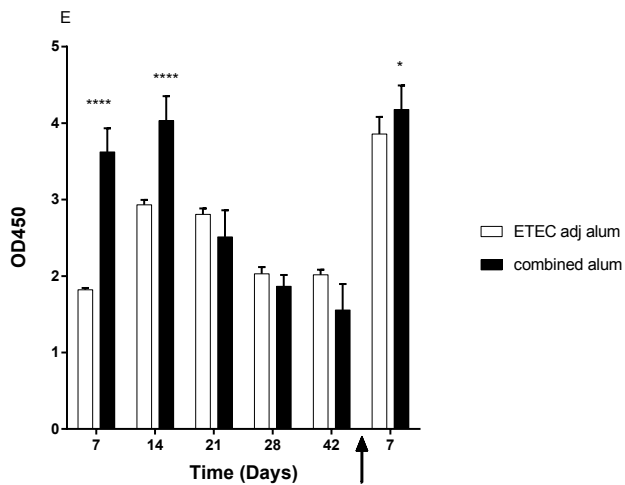

Supplement: Supplementary file 2 — 10.1186/s13104-016-1891-z Evaluation of in vivo specific IgG antibody response measured as absorbance values elicited by alum adjuvanted combined vaccine candidate. Balb/C mice (n = 10 mice per group) were immunized subcutaneously with 109 CFU of formalin killed whole cell antigens. Antigens belonged to the above-mentioned five-diarrheagenic E. coli pathotypes. Combined vaccine candidate consisted of formalin-killed whole cell of the main five pathotypes. Post-immunization blood samples were collected from mice groups weekly for six weeks. At week seven, mice were challenged with 106 CFU intraperitoneally and blood samples were collected one week after the challenge. Absorbance value of specific IgG antibody was measured for all seven intervals. Antibody absorbance values of combined vaccine candidate at selected time points compared to A) EAEC antigens, B) EPEC antigens, C) EIEC antigens, D) EHEC antigens and E) ETEC antigens. p < 0.05, ***p < 0.0001, and ****p < 0.00001, each bar represents mean ± standard deviation. [file 13104_2016_1891_MOESM2_ESM.pdf]

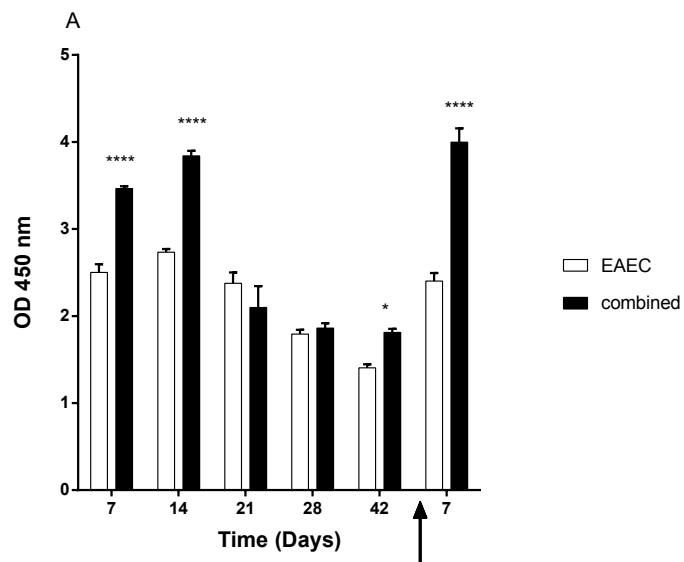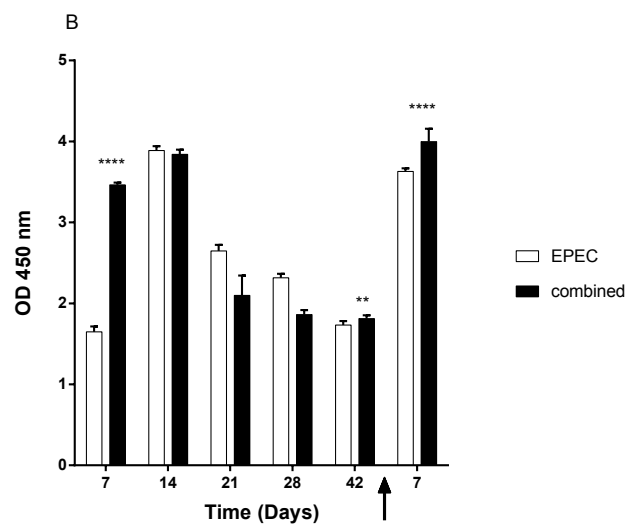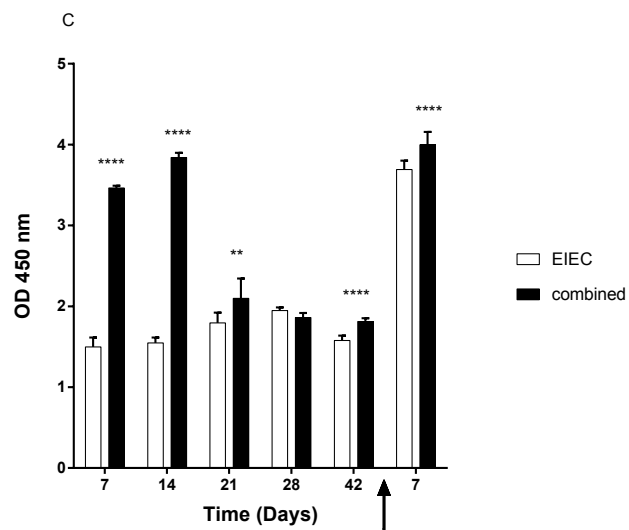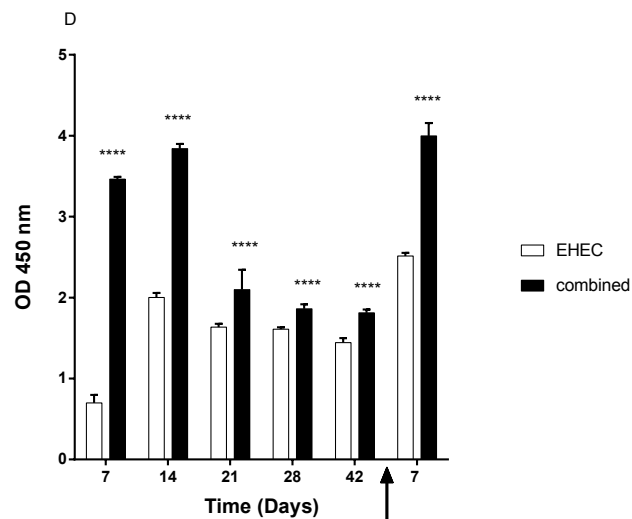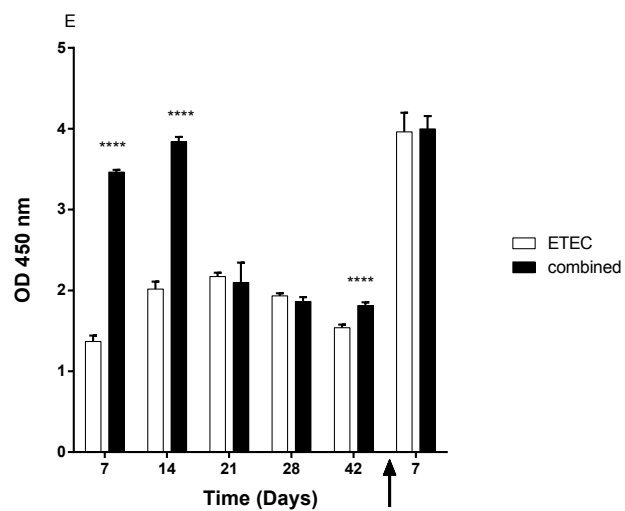

Supplement: Supplementary file 3 — 10.1186/s13104-016-1891-z Evaluation of in vivo specific IgG antibody response measured as absorbance values elicited by unadjuvanted combined vaccine candidate. Balb/C mice (n = 10 mice per group) were immunized subcutaneously with 109 CFU of formalin killed whole cell antigens. Antigens belonged to the above-mentioned five-diarrheagenic E. coli pathotypes. Combined vaccine candidate consisted of formalin-killed whole cell of the main five pathotypes. Post-immunization blood samples were collected from mice groups weekly for six weeks. At week seven, mice were challenged with 106 CFU intraperitoneally and blood samples were collected one week after the challenge. Absorbance values of specific IgG antibody were measured for all seven intervals. Antibody absorbance values of combined vaccine candidate at selected time points compared to A) EAEC antigens, B) EPEC antigens, C) EIEC antigens, D) EHEC antigens and E) ETEC antigens. (*p < 0.05, ** p < 0.001, ***p < 0.0001, and ****p < 0.00001), each bar represents mean ± standard deviation. [file 13104_2016_1891_MOESM3_ESM.pdf]

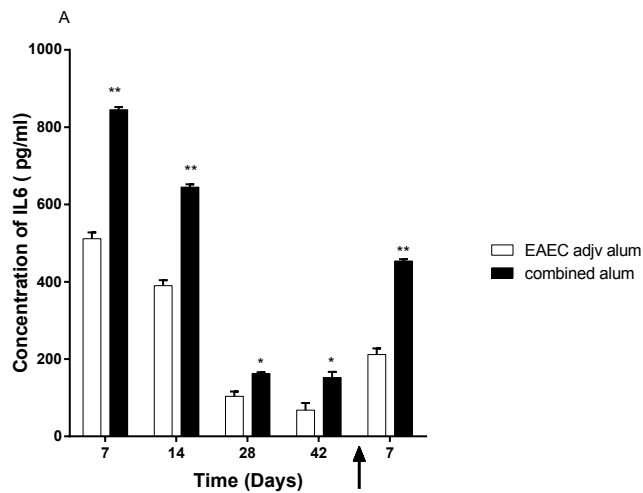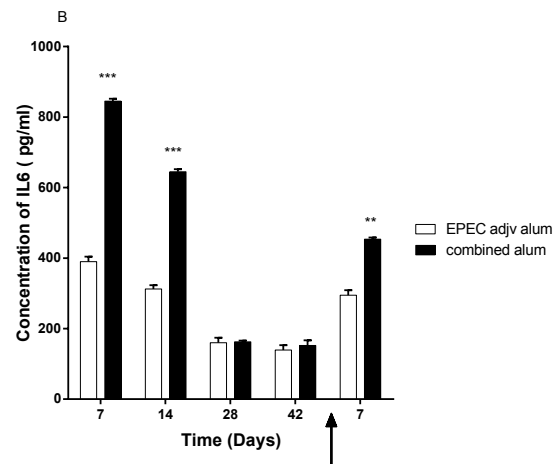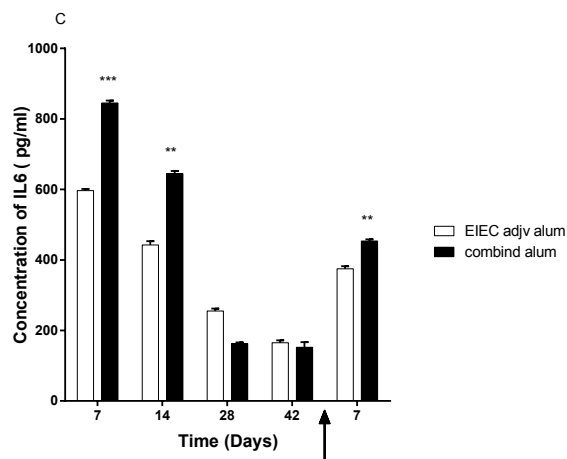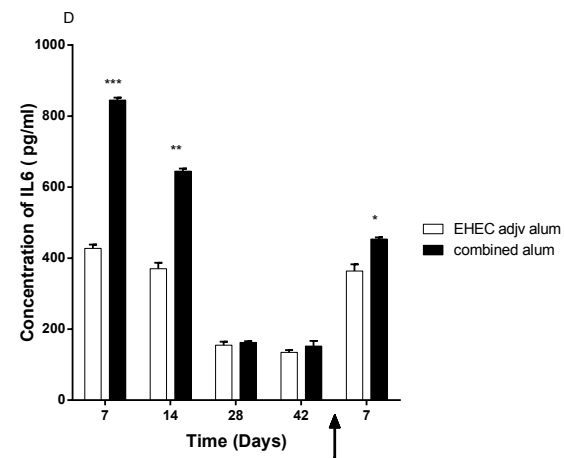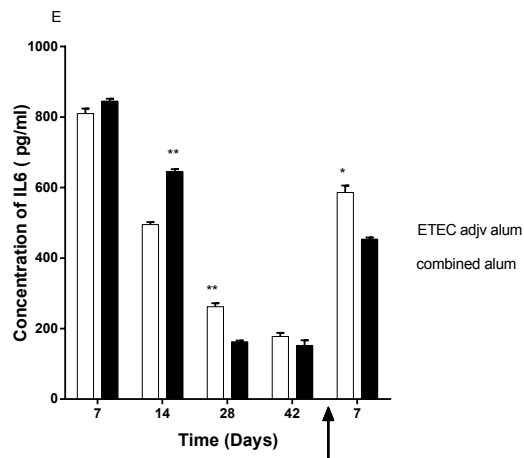

Supplement: Supplementary file 4 — 10.1186/s13104-016-1891-z Evaluation of IL-6 levels elicited by alum adjuvanted combined vaccine candidate. Balb/C mice (n = 10 mice per group) were immunized subcutaneously with 109 CFU of formalin killed whole cell antigens. Antigens belonged to the above-mentioned five-diarrheagenic E. coli pathotypes. Combined vaccine candidate consisted of formalin-killed whole cell of the main five pathotypes. Post-immunization blood samples were collected from mice groups weekly for six weeks. At week seven, mice were challenged with 106 CFU intraperitoneally and blood samples were collected one week after the challenge. The concentration of IL-6 was measured for all seven intervals. IL-6 concentration of combined vaccine candidate at selected time points compared to A) EAEC antigens, B) EPEC antigens, C) EIEC antigens, D) EHEC antigens and E) ETEC antigens. *p < 0.05, ** p < 0.001, and ***p < 0.0001 each bar represents mean ± standard deviation. [file 13104_2016_1891_MOESM4_ESM.pdf]

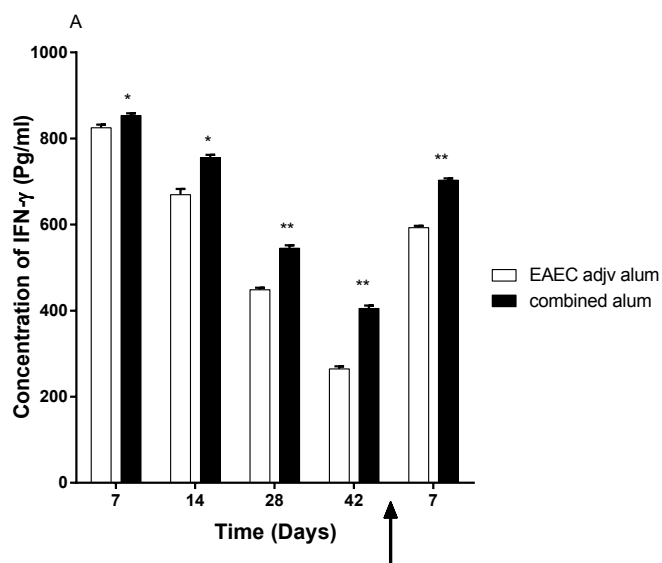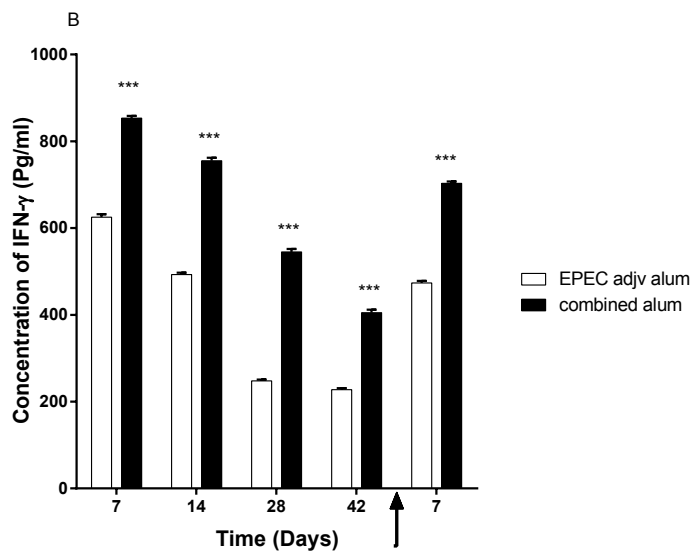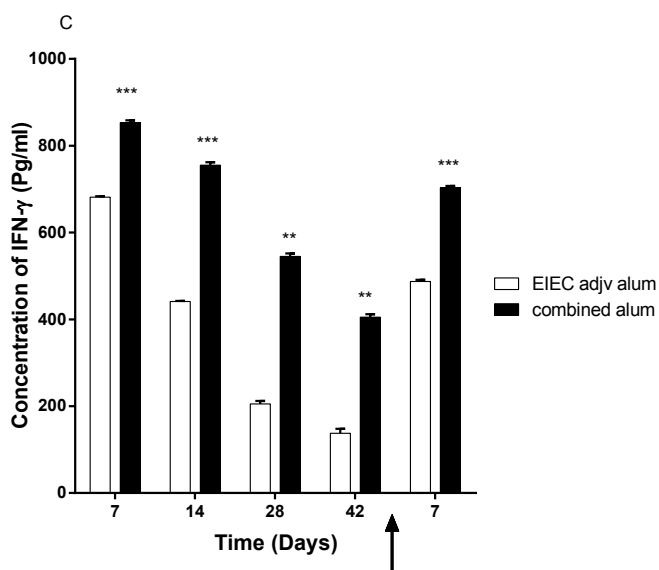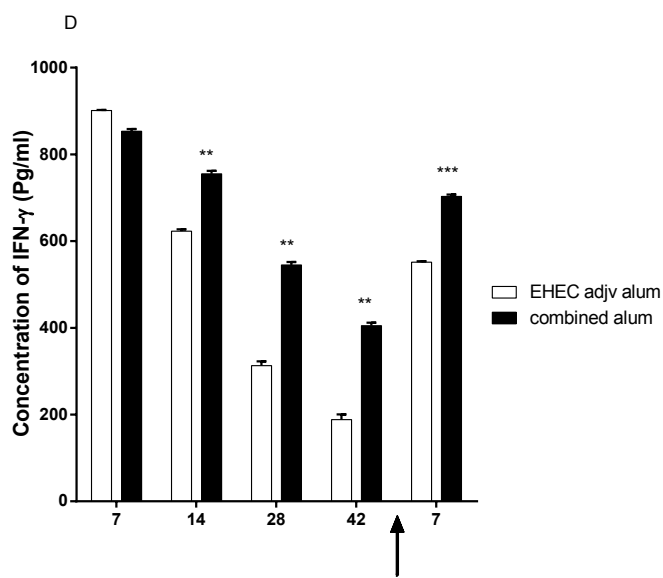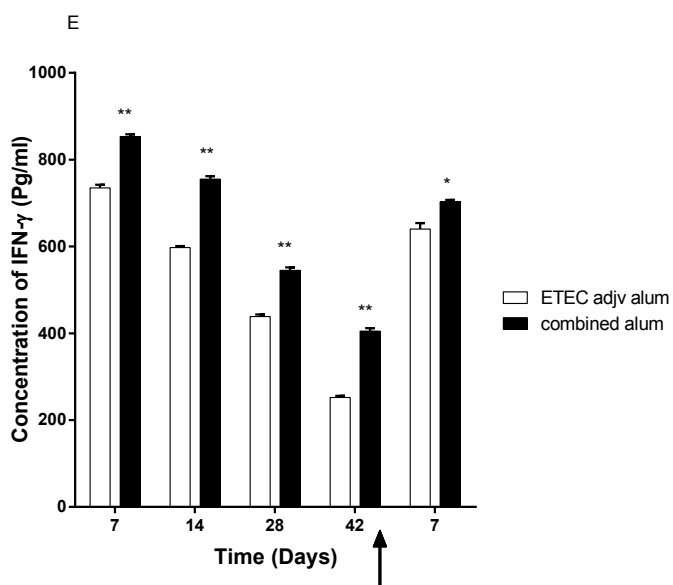

Supplement: Supplementary file 5 — 10.1186/s13104-016-1891-z Evaluation of IFNγ levels elicited by alum adjuvanted combined vaccine candidate. Balb/C mice (n = 10 mice per group) were immunized subcutaneously with 109 CFU of formalin killed whole cell antigens. Antigens belonged to the above-mentioned five-diarrheagenic E. coli pathotypes. Combined vaccine candidate consisted of formalin-killed whole cell of the main five pathotypes. Post-immunization blood samples were collected from mice groups weekly for six weeks. At week seven, mice were challenged with 106 CFU intraperitoneally and blood samples were collected one week after the challenge. The concentration of IFNγ was measured for all seven intervals. IFNγ concentration of combined vaccine candidate at selected time points compared to A) EAEC antigens, B) EPEC antigens, C) EIEC antigens, D) EHEC antigens and E) ETEC antigens.*p < 0.05, ** p < 0.001, and ***p < 0.0001, each bar represents mean ± standard deviation. [file 13104_2016_1891_MOESM5_ESM.pdf]

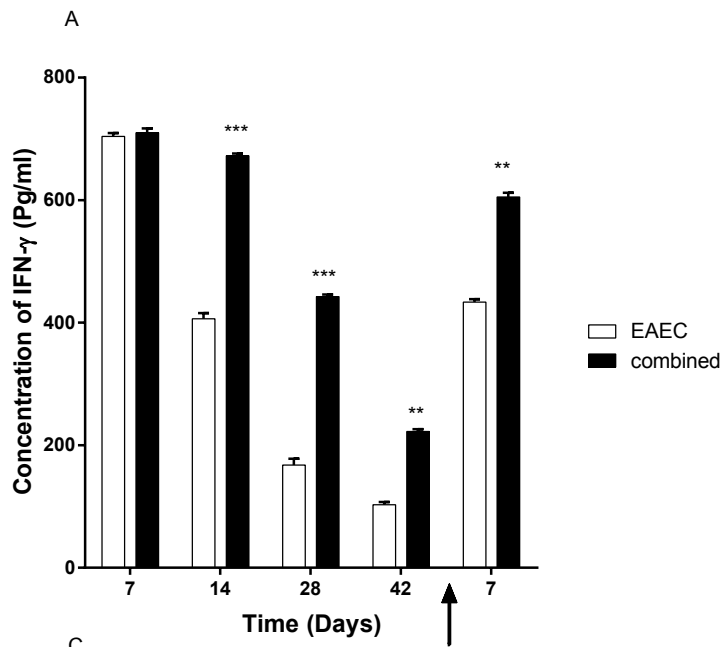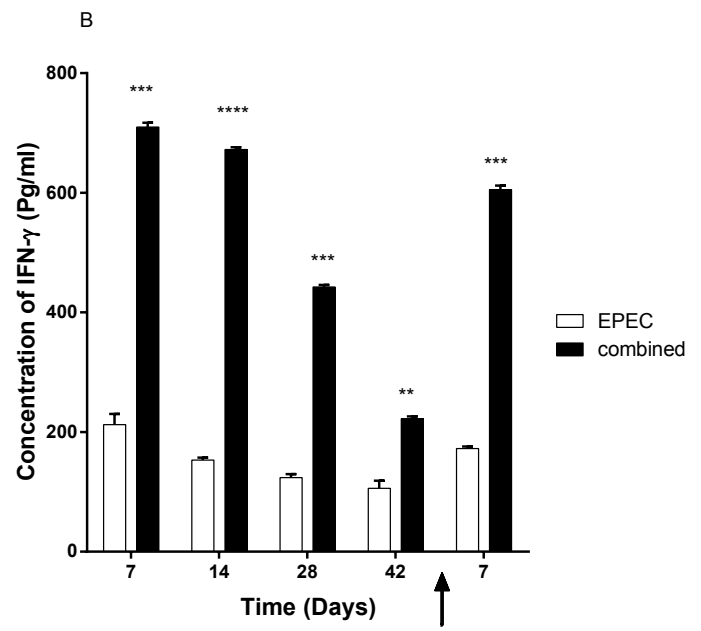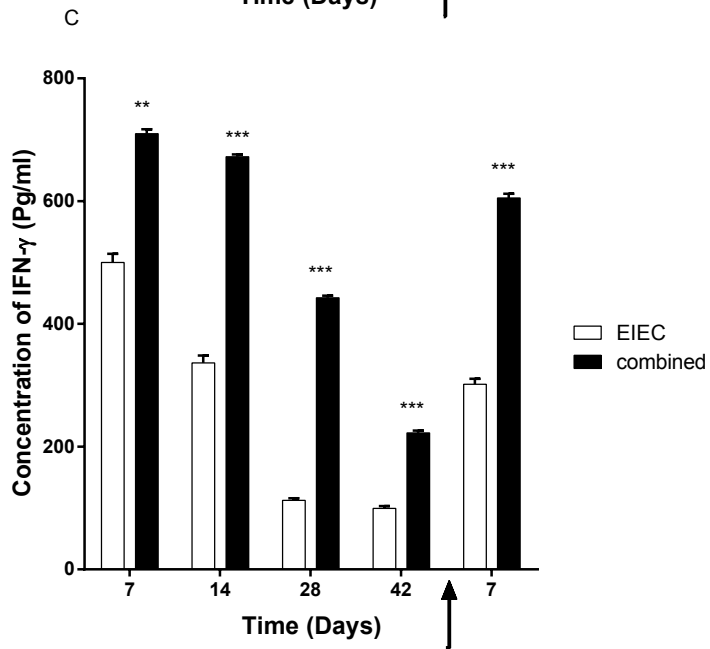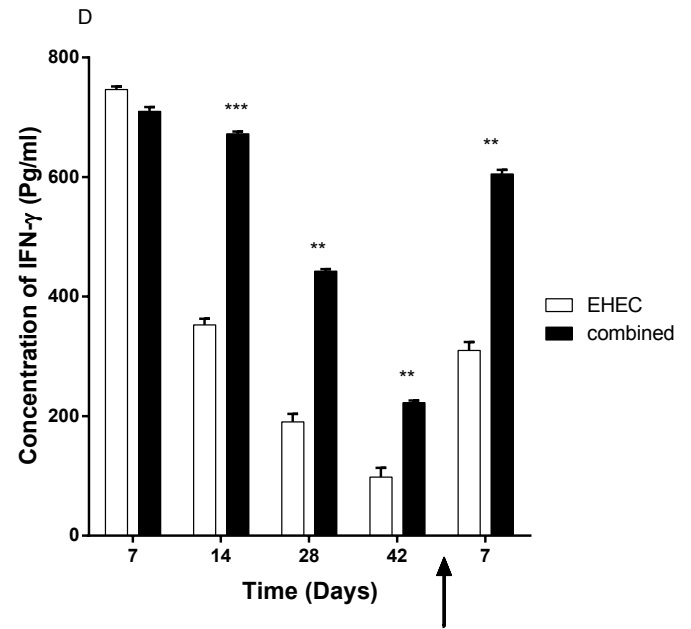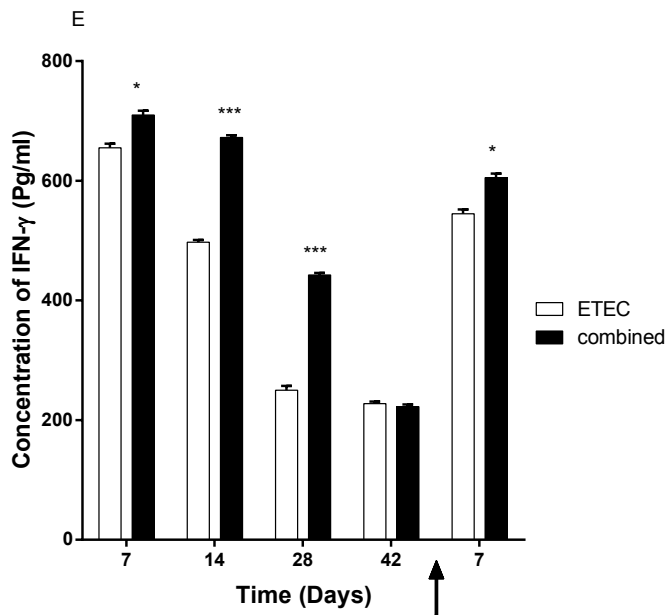

Supplement: Supplementary file 6 — 10.1186/s13104-016-1891-z Evaluation of IFNγ levels elicited by unadjuvanted combined vaccine candidate. Balb/C mice (n = 10 mice per group) were immunized subcutaneously with 109 CFU of formalin killed whole cell antigens. Antigens belonged to the above-mentioned five-diarrheagenic E. coli pathotypes. Combined vaccine candidate consisted of formalin-killed whole cell of the main five pathotypes. Post-immunization blood samples were collected from mice groups weekly for six weeks. At week seven, mice were challenged with 106 CFU intraperitoneally and blood samples were collected one week after the challenge. The concentration of IFNγ was measured for all seven intervals. IFNγ concentration of combined vaccine candidate at selected time points compared to A) EAEC antigens, B) EPEC antigens, C) EIEC antigens, D) EHEC antigens and E) ETEC antigens. *p < 0.05, ** p < 0.001, and ***p < 0.0001, each bar represents mean ± standard deviation. [file 13104_2016_1891_MOESM6_ESM.pdf]

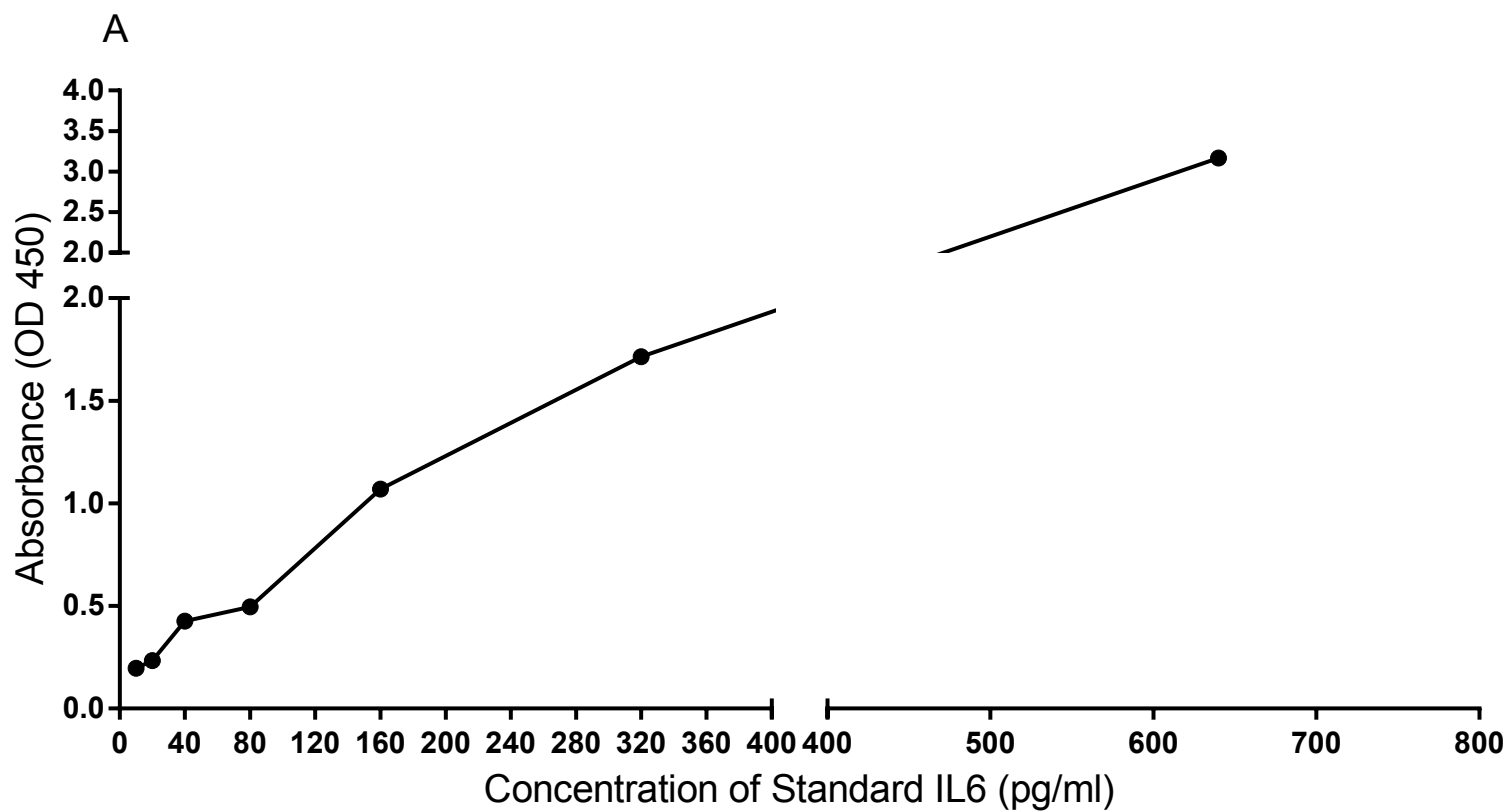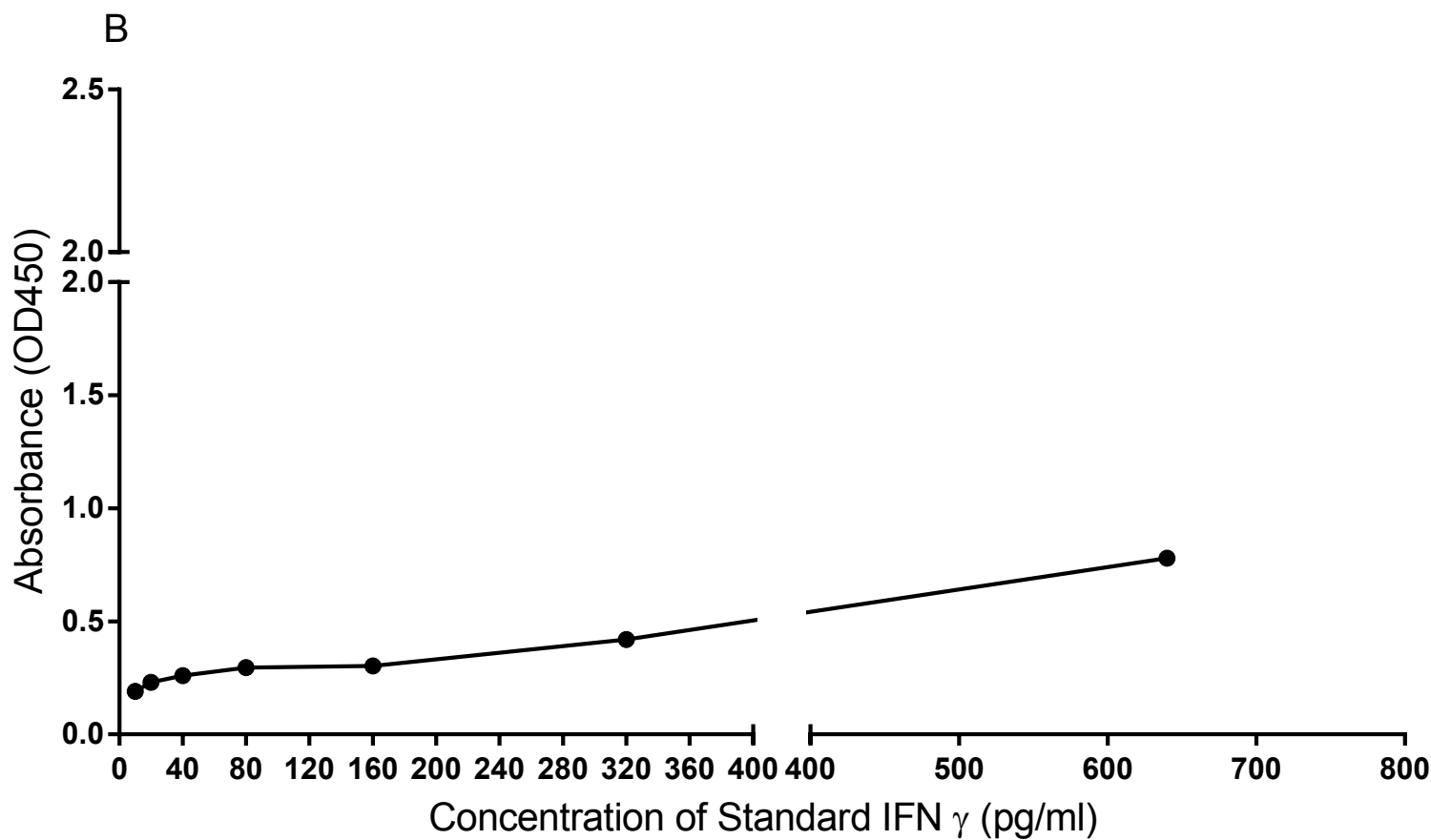

Supplement: Supplementary file 7 — 10.1186/s13104-016-1891-z Standard curve of IL-6 and IFNγ in pg/ml. [file 13104_2016_1891_MOESM7_ESM.pdf]
